# Modelling the pathology and treatment of cardiac fibrosis in vascularised atrial and ventricular cardiac microtissues

**DOI:** 10.3389/fcvm.2023.1156759

**Published:** 2023-09-01

**Authors:** Jasmeet S. Reyat, Alessandro di Maio, Beata Grygielska, Jeremy Pike, Samuel Kemble, Antonio Rodriguez-Romero, Christina Simoglou Karali, Adam P. Croft, Bethan Psaila, Filipa Simões, Julie Rayes, Abdullah O. Khan

**Affiliations:** ^1^ College of Medical and Dental Sciences, Institute of Cardiovascular Sciences, University of Birmingham, Birmingham, United Kingdom; ^2^ Department of Physiology, Anatomy and Genetics, Institute of Developmental and Regenerative Medicine, University of Oxford, Oxford, United Kingdom; ^3^ The Centre of Membrane Proteins and Receptors (COMPARE), University of Birmingham and University of Nottingham, Birmingham, United Kingdom; ^4^ Rheumatology Research Group, College of Medical and Dental Sciences, Institute of Inflammation and Ageing, University of Birmingham, Queen Elizabeth Hospital, Birmingham, United Kingdom; ^5^ Radcliffe Department of Medicine and National Institute of Health Research (NIHR) Oxford Biomedical Research Centre, MRC Weatherall Institute of Molecular Medicine, University of Oxford, Oxford, United Kingdom; ^6^ Cancer and Haematology Centre, Churchill Hospital, Oxford University Hospitals NHS Foundation Trust, Oxford, United Kingdom

**Keywords:** 3D cardiac microtissues, induced pluripotent stem cells, tissue engineering, cardiac fibrosis, cardiomyocytes

## Abstract

**Introduction:**

Recent advances in human cardiac 3D approaches have yielded progressively more complex and physiologically relevant culture systems. However, their application in the study of complex pathological processes, such as inflammation and fibrosis, and their utility as models for drug development have been thus far limited.

**Methods:**

In this work, we report the development of chamber-specific, vascularised human induced pluripotent stem cell-derived cardiac microtissues, which allow for the multi-parametric assessment of cardiac fibrosis.

**Results:**

We demonstrate the generation of a robust vascular system in the microtissues composed of endothelial cells, fibroblasts and atrial or ventricular cardiomyocytes that exhibit gene expression signatures, architectural, and electrophysiological resemblance to *in vivo*-derived anatomical cardiac tissues. Following pro-fibrotic stimulation using TGFβ, cardiac microtissues recapitulated hallmarks of cardiac fibrosis, including myofibroblast activation and collagen deposition. A study of Ca^2+^ dynamics in fibrotic microtissues using optical mapping revealed prolonged Ca^2+^ decay, reflecting cardiomyocyte dysfunction, which is linked to the severity of fibrosis. This phenotype could be reversed by TGFβ receptor inhibition or by using the BET bromodomain inhibitor, JQ1.

**Discussion:**

In conclusion, we present a novel methodology for the generation of chamber-specific cardiac microtissues that is highly scalable and allows for the multi-parametric assessment of cardiac remodelling and pharmacological screening.

## Introduction

Cardiovascular diseases (CVDs) remain the leading cause of death in the western world (1–3), with current avenues in the development of novel cardiovascular therapeutics yielding poor outcomes due to inherent adverse side effects (4). As a result, there is an unmet need to develop safer and more effective, reproducible and high throughput cardiac models to assess the efficacy of new therapies to improve pre-clinical research (4). While recent cell line-based technologies provide some insight into how human CVDs can be modelled *in vitro*, they fail to reproduce the complexity of the native system (5). Although small animal models have been extensively used to model CVDs, there remain inherent differences in physiology which can confound attempts to translate these studies to human disease and therapies (6).

The use of human induced pluripotent stem cells (hiPSCs) has provided a means for the generation of cardiomyocytes for personalised disease modelling and drug screening (5, 7–10). These cells have been effective in understanding cardiac development, as well cardiac disease modelling and toxicology. However, thus far these approaches have had limited direct translational application as hiPSC-derived cardiomyocytes are embryonic, lacking the architecture, functionality, and molecular composition of adult/primary cardiomyocytes (11). Moreover, 2D single-cell type cultures do not effectively recapitulate the cellular heterogeneity present in the cardiac tissue *in vivo*. Therefore, the study of more complex and difficult to treat pathologies like cardiac fibrosis in these models is limited due to the lack of intercellular interactions, which contribute to the onset and progression of disease.

Recent advances in cardiac tissue engineering technologies have allowed the development of physiologically relevant tissue models, that emulate a higher degree of complexity, organisation and dynamics that is seen in human cardiac tissue (12–14). These include hydrogel-cell mixtures, whereby multiple cell types (e.g., cardiomyocytes, endothelial cells and fibroblasts) are encapsulated into hydrogel structures to generate 3D bioprinted tissues (15–19). Cardiac sheets and engineered heart tissues involve the growth of cardiomyocytes in addition to other cell types in collagen moulds, which can be used as cardiac patches for cardiac regeneration (20, 21). In addition, 3D cardiac microtissue models (also known as cardiac organoids) have emerged, including both self-assembling and bioprinted structures (22–24).

Cardiac microtissues were first described using murine pluripotent stem cells, or as part of gastruloids, in which generated cells of interest spontaneously self-organised with distinct atrial and ventricular like regions (25, 26). Following on from these studies, similar models were generated using hiPSCs by assembling different cardiac cell types (15, 18, 19, 23, 27–36), and more recently self-organising models using pre-differentiated hiPSCs that recapitulate cardiac development (16, 17, 26, 37–41). Both types of cardiac microtissue models offer significant advances over standard 2D culture techniques and those involving advanced tissue engineering approaches with improved cell-cell communication between cardiomyocytes and other cell types while using fewer cells than other tissue engineering approaches (42). In addition, many of these microtissue models have substantially relied on the use of ventricular cardiomyocytes, the use of external extracellular matrix compositions, such as Matrigel, or the co-emergence of cardiac microtissues with gut tissue (15, 18, 19, 23, 27–38, 43, 44). Importantly, the extracellular matrix plays a role in regulating mechanical stress and supporting vasculogenesis (45). Several cardiac microtissue models have been described incorporating either synthetic or natural materials like collagen and Matrigel (16, 17, 19). Although these microtissue models incorporated endothelial cells either through direct differentiation protocols or through the addition of endothelial cells from human sources, they lack the architectural organisation that supports cellular crosstalk. For example, microtissue models reporting an endothelial compartment don't show the patterned branched vessel network observed in the heart which is important for the alignment of axillary stromal and mural cells. Finally, studies utilising self-assembling microtissues have primarily focused on the developmental aspects of cardiac microtissue generation (16, 17, 39–41), with thus far limited attention to the utility of complex 3D culture systems for modelling late-onset multi-cellular pathologies and interrogating potential pharmacological interventions.

Here we report a new microtissue culture method by differentiating vascular and cardiomyocyte lineages (atrial and ventricular) from hiPSCs before combing them to generate chamber-specific cardiac microtissues. This approach scalably and reproducibly generates vascularised atrial- and ventricular-specific cardiac microtissues, which were validated transcriptionally, architecturally and functionally using optical mapping electrophysiology. When challenged with transforming growth factor-β (TGFβ) to mimic acute effects of cardiac fibrosis (46), these multicellular human cardiac microtissues recapitulated the progressive nature of cardiac fibrosis pathology, including fibroblast activation, fibroblast to myofibroblast transition, and excessive collagen deposition. Optical mapping demonstrated a functional effect of fibrosis, and blockade of these phenotypes could be observed using the TGFβ receptor inhibitor SB431542, and the BET bromodomain inhibitor JQ1. Our results highlight the generation of robust vascularised chamber-specific cardiac models that can be used as an effective platform for drug discovery and *in vitro* disease modelling.

## Results

### Generation of atrial and ventricular cardiac microtissue models from human iPSCs

**Figure 1:**
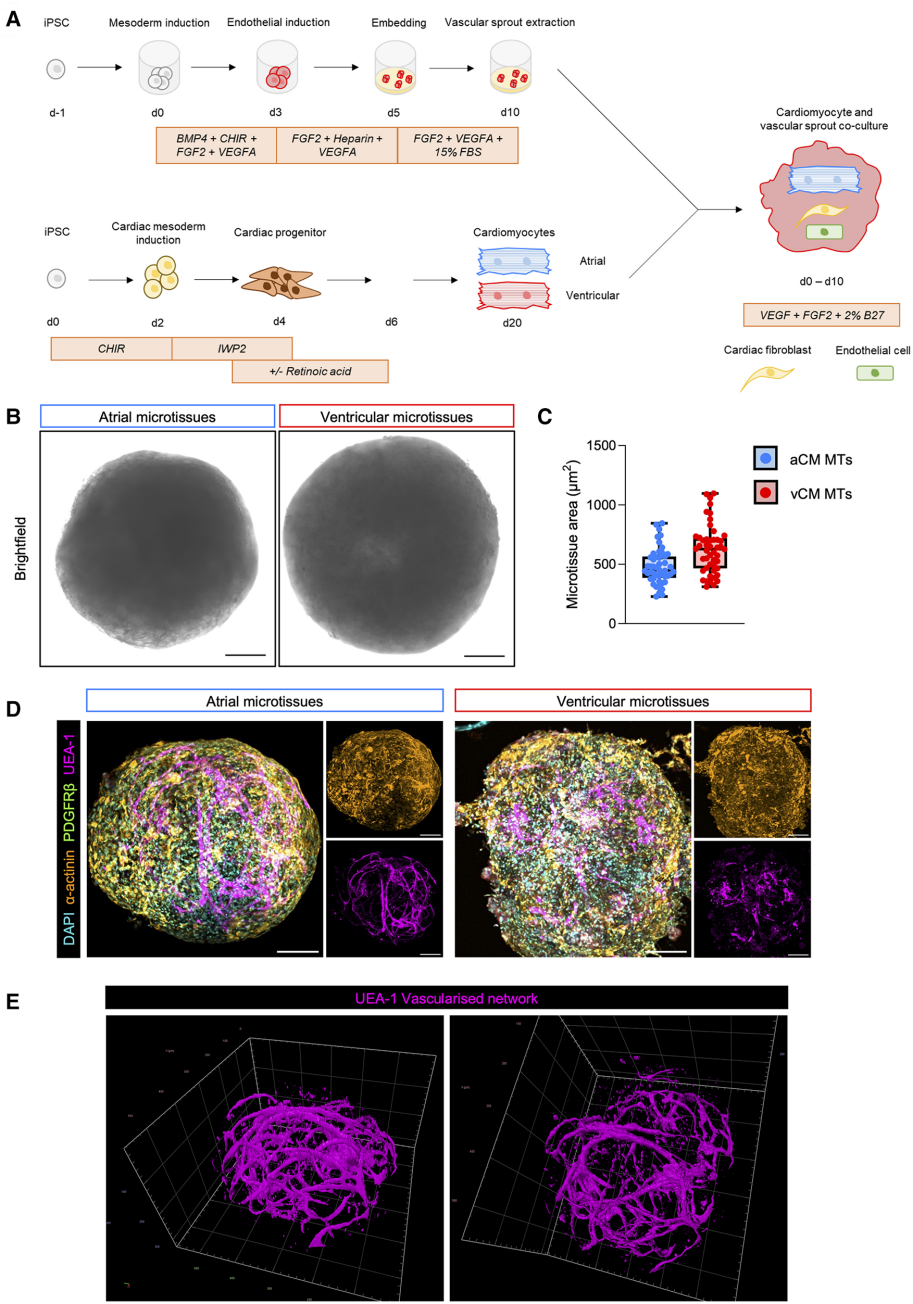
Generation of multicellular chamber-specific human iPSC-derived cardiac microtissues. (**A**) Schematic representation of atrial (aCM MT) and ventricular microtissue (vCM MT) generation methodology. (**B**) Representative bright-field images of 3D day 10 aCM MT and vCM MT. The scale bar represents 100 µm. (**C**) Quantification of aCM MTs and vCM MTs size. Data are presented as mean ± SD (*n* = 48 microtissues from 6 independent experiments). (**D**) Whole microtissue immunofluorescent staining of α-actinin (cardiomyocytes), PDGFRβ (fibroblasts), UEA-1 (endothelial cells) and DAPI (nuclei) in an aCM MT (left) and vCM- MT (right) at day 10. Smaller panels show single stained images of cardiomyocytes (α-actinin) and endothelial cells (UEA-1) showing cellular localisation within the cardiac microtissues. The scale bar represents 100 µm. (**E**) IMARIS rendered imaging of the endothelial vascularised network in the cardiac tissue.

While some emerging cardiac organoid models demonstrate endothelial differentiation, developed branching networks with supporting stromal compartments throughout the volume of 3D cardiac cultures have not yet been reported. Here we adapted an approach whereby vascular sprouts are generated through hydrogel embedding to generate a vascular scaffold (as reported by Wimmer et al.) onto which hiPSC-derived cardiomyocytes generated from parallel atrial and ventricular differentiations were seeded (47, 48) (Figure 1A). We exposed a common mesodermal aggregate to FGF2, VEGFA and embedded it in a collagen I:matrigel hydrogel to generate 3D vascular sprouts. These vascular sprouts expressed high levels of endothelial cell genes (*CDH5*, *ITGA4* and *PECAM1*) and fibroblast genes (*NG2*, *PDGFRB*, *COL1A2*, *ACTA2*, *POSTN* and *COL1A1*), but little to no expression of cardiomyocyte genes (*NKX2-5*, *ACTN2*, *IRX4*, *NPPA*, *TNNT2* and *MYL3*; Supplementary Figure S1). In parallel, chamber-specific cardiomyocytes were generated through the stepwise activation and inhibition of Wnt signalling (49) (Figure 1A).

To specify atrial-like cardiomyocytes, we added retinoic acid (RA) to the cultures from day 3 to 6. We observed beating cultures of atrial and ventricular cardiomyocytes as early as day 8 of differentiation and we cultured these until day 20. Cardiomyocyte atrial and ventricular differentiations yielded a high level of purity as assessed by flow cytometry using the cardiac marker cardiac troponin-T (Supplementary Figure S2A). Assessment of chamber-specificity of day 20 cardiomyocytes revealed robust expression of *MYL2* and *GJA1* in ventricular cardiomyocytes and *NR2F2*, *NPPA*, *KCNA5* and *CACNA1C* in atrial cardiomyocytes, consistent with previous reports of hiPSC-derived chamber-specific cardiomyocytes (49, 50) (Supplementary Figure S2B). Next, we dissociated atrial and ventricular cardiomyocytes into a single-cell suspension and co-cultured these with vascular sprouts in ultra-low attachment plates to facilitate cardiomyocyte clumping when forming cardiac microtissues.

We cultured the microtissues for an additional 10 days to promote synchronous beating across the microtissue. Brightfield image analysis revealed similar sizes of ventricular cardiac microtissues (vCM MTs) compared to atrial cardiac microtissues (aCM MTs) (Figures 1B,C). We have, therefore, established a new *in vitro* human chamber-specific vascularised cardiac microtissue model containing endothelial and fibroblasts, in addition to atrial or ventricular cardiomyocytes.

### Phenotypic characterisation of hiPSC-derived atrial and ventricular cardiac microtissues

To gain insight into the cellular organisation of cells within the cardiac microtissues, we carried out whole mount immunofluorescence analysis using cell-type specific markers. Both aCM MTs and vCM MTs showed expression of cardiomyocytes (α-actinin^+^) throughout the microtissues, in close proximity to endothelial cells (UEA1^+^) and fibroblasts (PDGRFβ^+^) (Figure 1D). Both MTs formed a compact spherical culture, with cardiomyocytes and fibroblasts amongst a core containing a vascular network of endothelial cells (Figure 1D). Further analysis of the endothelial compartment within the cardiac microtissues revealed a vascularised network investing the culture (Figure 1E).

**Figure 2:**
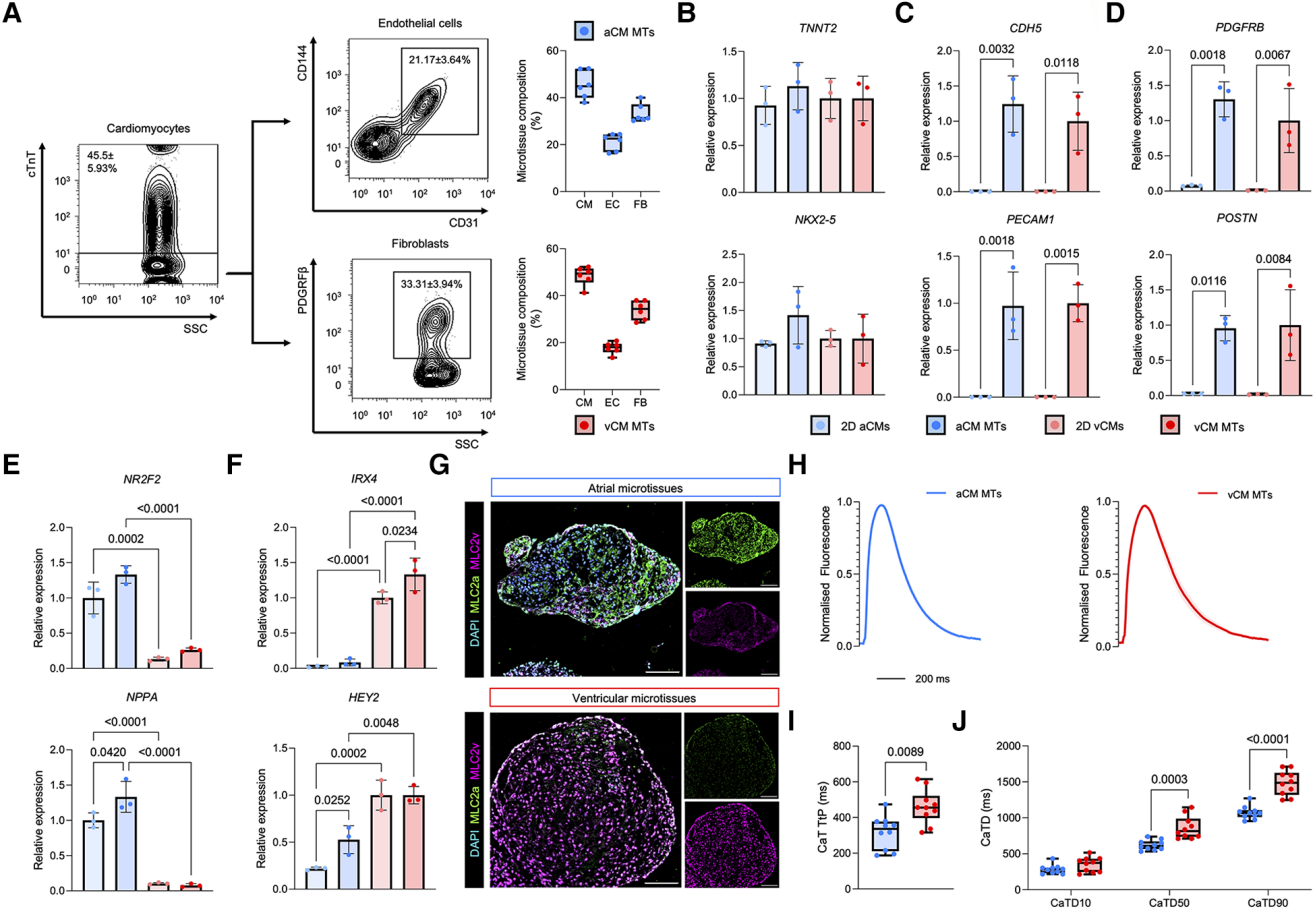
Cellular phenotyping of human iPSC-derived aCM MTs and vCM MTs reveals chamber specific cellular expression profiles. (**A**) Quantification of cardiomyocyte, endothelial and fibroblast populations in aCM MTs and vCM MTs by flow cytometry. Representative FACS plots are shown with the percentage microtissue composition quantification on the right. Data are presented as mean ± SD (*n* = 6 independent experiments). (**B–D**) Gene expression of (**B**) cardiomyocyte (*TNNT2* and *NKX2-5*), (**C**) endothelial (*CDH5* and *PECAM1*) and (**D**) fibroblast (*PDGFRβ* and *POSTN*) markers in day 10 aCM MTs and vCM MTs compared to 2D cultured day 20 hiPSC-derived aCMs and vCMs. Data are presented as mean ± SD (*n* = 3 independent experiments) relative to the expression of 2D hiPSC-vCMs in the case of cardiomyocyte genes or relative to 3D vCM MTs in the case of endothelial and fibroblast genes. Statistical analysis was performed using a One-way ANOVA followed by a Kruskal Wallis *post-hoc* test. (**E,F**) Gene expression of (**E**) atrial (*NR2F2* and *NPPA*) and (**F**) ventricular (*IRX4* and *HEY2*) in day 10 aCM MTs and vCM MTs compared to 2D cultured day 20 iPSC-derived aCMs and vCMs. Data are presented as mean ± SD (*n* = 3 independent experiments) relative to 2D hiPSC-aCMs for atrial genes or 2D hiPSC-vCMs for ventricular genes. Statistical analysis was performed using a One-way ANOVA followed by a Kruskal Wallis *post-hoc* test. (**G**) Immunofluorescence analysis of cardiac chamber specific myosin light chain variants (MLC2a and MLC2v) in day 10 sectioned aCM MTs and vCM MTs. The scale bar represents 100 µm. (**H–J**) Ca^2+^ dynamics in aCM MTs and vCM MTs using optical mapping. (**H**) Averaged Ca^2+^ traces from aCM MTs and vCM MTs. Error bars represent SEM. (**I**) Quantification of Ca^2+^ transient time-to-peak (CaT TtP) and (**J**) Ca^2+^ transient duration (CaTD) in aCM MTs and vCM MTs. Data are presented as mean ± SD (*n* = 10 aCM MTs and *n* = 10 vCM MTs from 3 independent experiments). Statistical analysis was performed using a Mann-Whitney *U*-test or a Two-way ANOVA when comparing between multiple groups.

Next, we performed quantitative fluorescent antibody-based profiling of the cardiac microtissues by flow cytometry with the cardiac marker cTnT, the endothelial markers CD31 and CD144 and the fibroblast marker PDGRFβ. aCM MTs and vCM MTs contained similar frequency expression patterns with 45.4 ± 5.93% cTnT^+^ cells, 21.17 ± 3.64% CD31^+^CD144^+^ cells and 33.31 ± 3.94% PDGFRβ^+^ cells (Figure 2A). Additional gene expression analysis showed similar expression of *TNNT2* and *NKX2-5* in aCM MTs and vCM MTs compared to 2D cultured hiPSC-derived atrial cardiomyocytes and ventricular cardiomyocytes (Figure 2B). In addition, the expression of endothelial genes *CDH5* and *PECAM1* (Figure 2C), along with fibroblast genes *PDGFRB* and *POSTN* (Figure 2D), was only observed in aCM MTs and vCM MTs at similar levels, but absent in standard CM 2D-cultures. Together, this data demonstrates that cardiac microtissues are comprised of a vascular network supporting cardiomyocytes and fibroblasts in a ratio comparable to adult human cardiac tissue (51, 52).

To investigate whether MT cardiomyocytes retained their chamber-specific gene signatures, we performed qRT-PCR and immunofluorescence imaging of atrial and ventricular specific cardiomyocyte markers (49). aCM MTs showed robust expression of the atrial markers *NR2F2* and *NPPA*, similar to 2D cultured atrial cardiomyocytes (Figure 2E), whilst the expression of these markers was low in vCM MTs and 2D cultured ventricular cardiomyocytes. On the contrary, vCM MTs expressed high levels of the ventricular markers *IRX4* and *HEY2*, which were expressed at lower levels in aCM MTs and 2D cultured atrial cardiomyocytes (Figure 2F). In addition, culture of the cardiac microtissues improved the expression of cardiac structural genes, including *TCAP* and *GJA1* that encode for the sarcomeric protein Titin-cap and the junctional protein Connexin 43, respectively (Supplementary Figure S3). In addition, incorporation of cardiomyocytes into the microtissue environment increased the expression of the adult isoform of cardiac troponin (*TNNI3*), with reduced expression of the fetal isoform (*TNNI1*), suggesting cardiomyocyte maturation (Supplementary Figure S3). Immunofluorescence staining of sectioned cardiac MTs revealed high expression of the atrial myosin light chain variant (MLC2a) in aCM MTs, whilst vCM MTs expressed high levels of the ventricular variant (Figure 2G). This data show MT-cardiac chamber specificity at both the RNA and protein level.

In the human heart, atrial and ventricular cardiomyocytes acquire unique electrophysiological properties that play an important functional role in their excitation-contraction coupling (53). Fundamental to this, is the regulation of Ca^2+^ within cardiomyocytes. To functionally characterise the Ca^2+^ dynamics within the cardiac microtissues, optical mapping was conducted using the Ca^2+^ indicator dye Rhod-2 AM (49). aCM MTs and vCM MTs showed differences in Ca^2+^ trace morphology, with the vCM MTs having a more prolonged Ca^2+^ trace compared to aCM MTs (Figure 2H and Supplementary Figure S4A). In addition, vCM MTs showed a slower time to peak (Figure 2I) and prolonged Ca^2+^ transient duration at CaTD50 and CaTD90 (Figure 2J) compared to aCM MTs. Additional calcium analysis revealed a prolonged calcium transient decay in vCM MTs compared to aCM MTs which was associated with no changes in Ca^2+^ tau nor the beating rate between aCM MTs and vCM MTs (Supplementary Figures S4B–D). This functional characterisation of Ca^2+^ dynamics reveals that aCM MTs display similar properties to cardiomyocytes from human atria, whereas vCM MTs show similarities to human ventricles. This data show that our protocol robustly incorporates hiPSC-derived atrial and ventricular cardiomyocytes to form vascularised 3D cardiac chamber-specific microtissues, which retain chamber specificity at the gene expression, protein and function level.

### Treatment with TGFβ drives fibrotic remodelling in atrial cardiac microtissues

**Figure 3:**
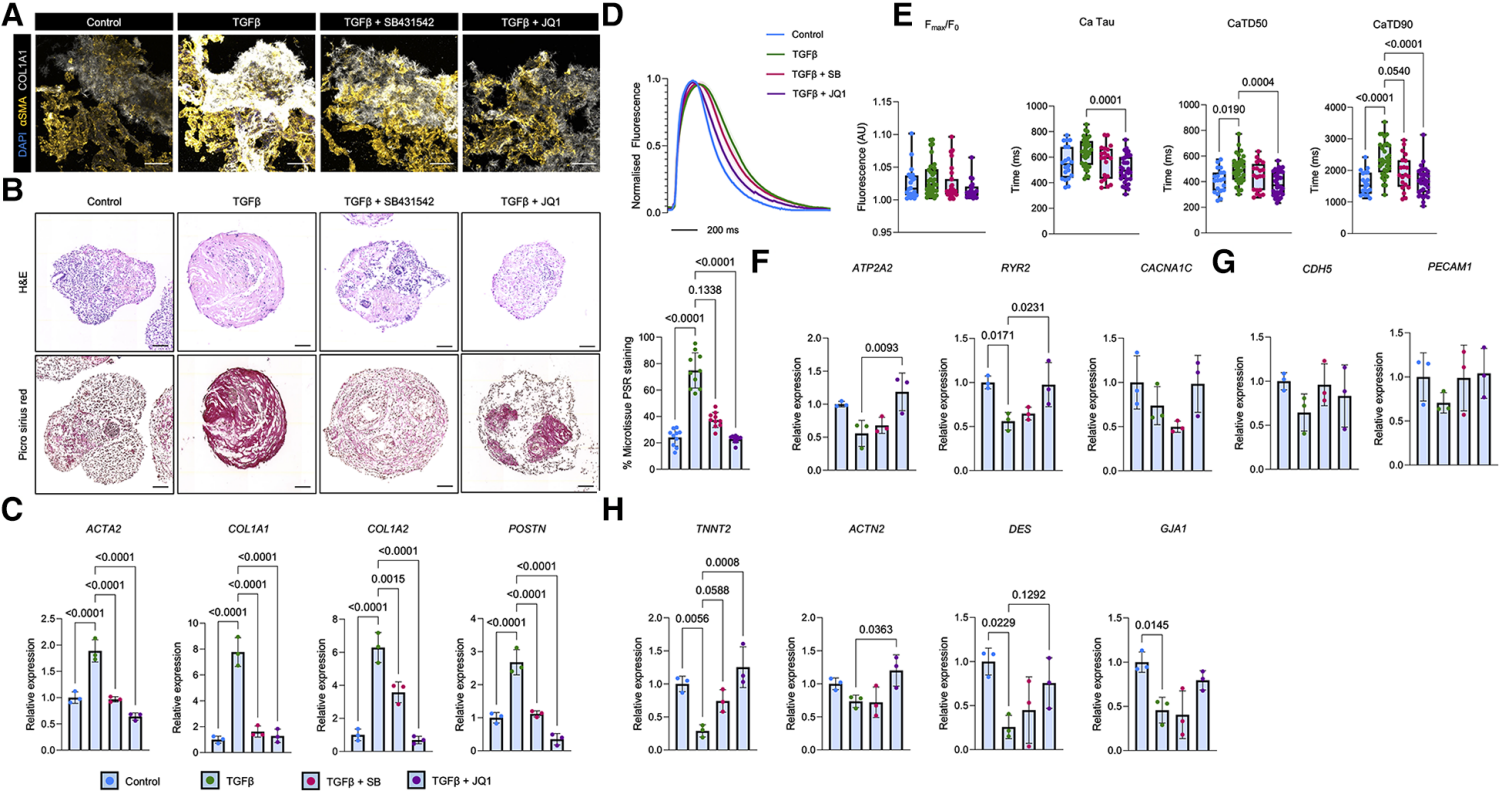
Modelling TGFβ-induced cardiac fibrosis in aCM MTs and its blockade using SB431542 and JQ1. (**A**) Immunofluorescent analysis of pro-fibrotic markers (αSMA and COL1A1) on sectioned aCM MTs following a 2 days treatment with TGFβ alone or in combination with a TGFβ-receptor I inhibitor (SB431542) or a BET bromodomain inhibitor (JQ1). The scale bar represents 100 µm. (**B**) Representative haematoxylin and eosin (H&E) and Picro Sirius Red stained images of aCM MTs treated with TGFβ alone or in combination with SB431542 or JQ1. Quantification of Picro Sirius Red is shown as a percentage of the total microtissue (right; *n* = 10 from 2 independent experiments). The scale bar represents 75 µm. (**C**) Gene expression analysis of pro-fibrotic genes (*ACTA2*, *COL1A1*, *COL1A2* and *POSTN*) in aCM MTs treated with TGFβ alone or in combination with SB431542 (SB) or JQ1. Data are presented as mean ± SD (*n* = 3 independent experiments) relative to control treated aCM-MTs. Statistical analysis was performed using a Kruskal-Wallis test. (**D,E**) Ca^2+^ dynamics in aCM MTs treated with TGFβ alone or in combination with SB431542 (SB) or JQ1. (**D**) Averaged Ca^2+^ traces of treated aCM MTs. Error bars represent SEM. (**E**) Quantification of Ca^2+^ time to peak (F_max_/F_0_), Ca^2+^ tau, Ca^2+^ transient duration 50% (CaTD50) and Ca^2+^ transient duration (CaTD). Data are presented as mean ± SD (*n* = 20 Control, *n* = 33 TGFβ, *n* = 32 TGFβ + SB and *n* = 20 TGFβ + JQ1 treated aCM MTs from 4 independent experiments). Statistical analysis was performed using a Kruskal-Wallis test. (**F**) Gene expression analysis of calcium handling genes (*ATP2A2*, *RYR2* and *CACNA1C*) in aCM MTs treated with TGFβ alone or in combination with SB or JQ1. Data are presented as mean ± SD (*n* = 3 independent experiments) relative to control treated aCM-MTs. Statistical analysis was performed using a Kruskal-Wallis test. (**G**) Gene expression analysis of endothelial (*CDH5* and *PECAM1*) and (**H**) Cardiac (*TNNT2*, *ACTN2*, *DES* and *GJA1*) and in aCM MTs treated with TGFβ alone or in combination with SB or JQ1. Data are presented as mean ± SD (*n* = 3 independent experiments) relative to control aCM-MTs. Statistical analysis was performed using a Kruskal-Wallis test.

In order to validate the functionality of the cardiac microtissues for disease modelling, we assessed the ability of the cardiac microtissues to mimic cardiac fibrosis as a model of cardiac dysfunction (54). To do this, we incubated aCM MTs with the potent pro-fibrotic stimulant TGFβ (46) and assessed the resultant fibrosis by immunofluorescence analysis of sectioned aCM MTs. We observed increased expression of α-smooth muscle actin (αSMA) and collagen 1 (COL1A1) (Figure 3A). Furthermore, TGFβ-induced fibrosis in aCM MTs was confirmed using haematoxylin and eosin (H&E) and Picro Sirius red staining (Figure 3B). At the gene level, TGFβ treatment promoted an increase in the expression of the pro-fibrotic genes *ACTA2*, *COL1A1*, *COL1A2* and *POSTN* in aCM MTs (Figure 3C). This effect was prevented by using SB431542, a selective small molecule inhibitor for TGFβ receptor I (55), and the BET bromodomain small molecule inhibitor JQ1, which has been shown to block cardiac fibrosis (56–58). In both cases, we observed reduced expression of αSMA and COL1A1 (Figure 3A), reduced collagen deposition (as shown by Picro Sirius red staining, Figure 3B) and reduced expression of key pro-fibrotic genes (*ACTA2*, *COL1A1*, *COL1A2* and *POSTN*, Figure 3C) in aCM MTs.

Recent work indicates that altered Ca^2+^ signalling in cardiomyocytes is observed during cardiac fibrosis, and that changes in Ca^2+^ dynamics can reflect optimal cardiomyocyte function (59, 60). Therefore, measuring changes in Ca^2+^ offers a potential readout for the evaluation of fibrotic severity, but also the efficacy of pharmacological interventions which may ameliorate fibrotic markers at the gene level, but with minimal effects on restoring function. We therefore employed optical mapping-based live cell imaging using the Ca^2+^ indicator, Rhod-2 AM, to quantify Ca^2+^ activity (49) in our cardiac microtissues subjected to pro-fibrotic stimulation. In aCM MTs, TGFβ treatment resulted in a prolonged Ca^2+^ trace (Figure 3D). This was accompanied by an increase in Ca^2+^ decay (Ca Tau) and Ca^2+^ transient duration (CaTD50 and CaTD90) without changing Ca^2+^ peak fluorescence (F_max_/F_0_) (Figure 3E).

We next assessed the effects of fibrosis blockade on Ca^2+^ dynamics. The combined treatment of TGFβ with JQ1, but not with SB431542, shortened the Ca^2+^ transient, with only TGFβ plus JQ1 treatment sufficient to reduce Ca^2+^ decay (Figures 3D,E). Additional Ca^2+^ transient analysis revealed the presence of ectopic Ca^2+^ spark events following TGFβ treatment which were absent in control, SB431542 or JQ1 treated aCM MTs (Supplementary Figure S5A). No changes in calcium time-to-peak nor beating rate were observed following TGFβ treatment in the presence or absence of TGFβ inhibitors (Supplementary Figure S5C). At the molecular level, TGFβ treatment in aCM MTs resulted in a reduction in *ATP2A2* and *RYR2* expression (Figure 3F). These genes encode the SERCA channel and the ryanodine receptor proteins, respectively, which are involved in cardiomyocyte Ca^2+^ handling. The expression levels of *ATP2A2* and *RYR2* could be restored to basal levels following JQ1 incubation, but not SB431542 treatment (Figure 3F). No changes in *CACNA1C*, that encodes the Ca^2+^ channel Cav1.2, were observed following TGFβ plus anti-fibrotic inhibitors treatment (Figure 3F).

Concerning additional cell types within aCM MTs, qRT-PCR analysis of TGFβ treated aCM MTs revealed no changes in the expression levels of endothelial genes (*CDH5* and *PECAM1*) (Figure 3G) but significant reductions in cardiac gene expression levels (*TNNT2*, *DES* and *GJA1*) (Figure 3H). Interestingly, TGFβ plus JQ1 treatment of aCM MTs restored the expression of *TNNT2*, *ACTN2*, *DES* and *GJA1*. Conversely, TGFβ plus SB431542 treatment only improved the expression of *TNNT2*, but not of the other cardiac genes (Figure 3H).

Taken together, this data suggests that TGFβ potently alters basal Ca^2+^ dynamics in aCM MTs, which is driven largely through changes in cardiomyocyte function. These phenotypes can be prevented by JQ1 treatment, but not SB431542 treatment.

### Modelling TGFβ-induced cardiac remodelling in ventricular cardiac microtissues

**Figure 4:**
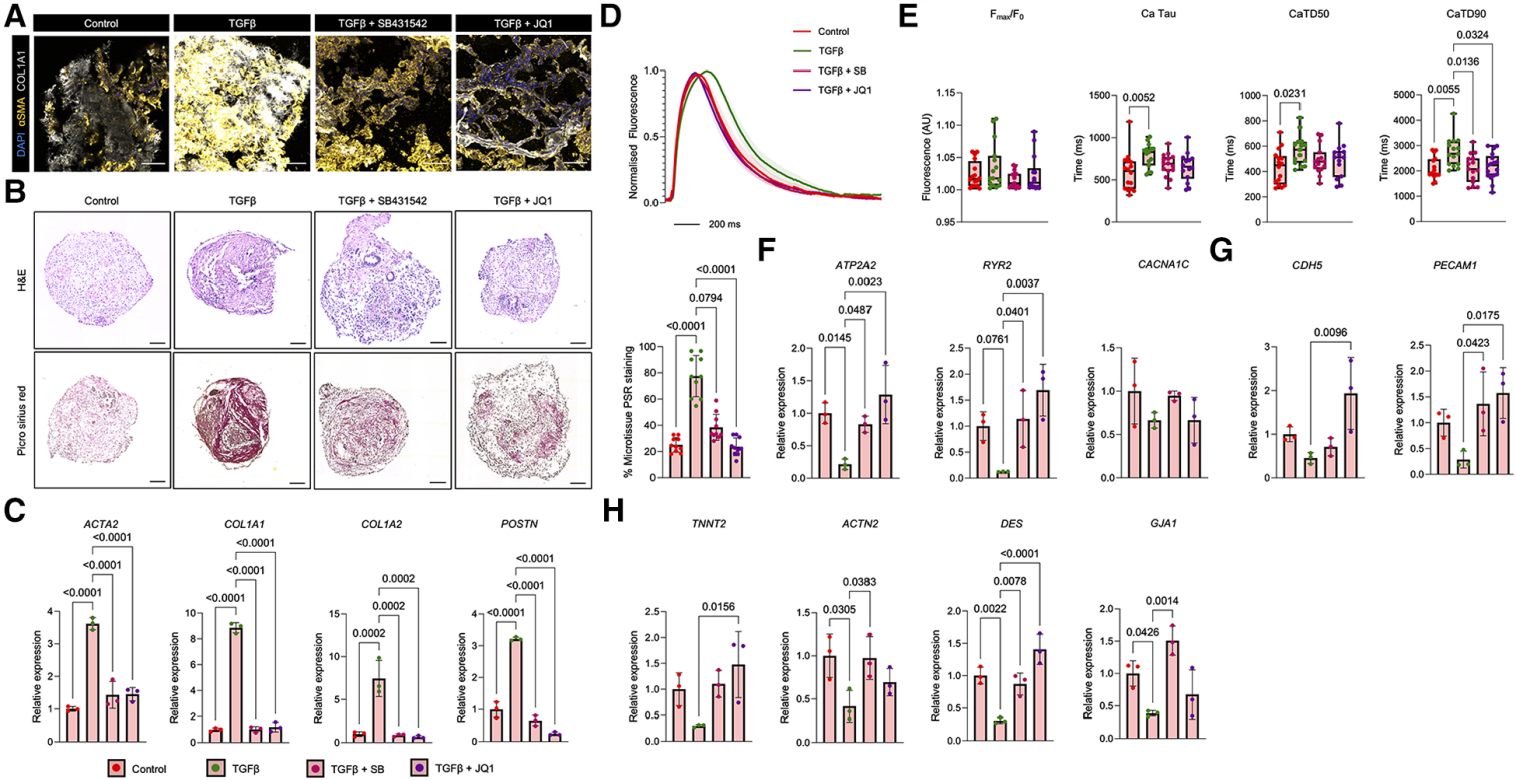
Fibrotic phenotyping of vCM MTs treated with TGFβ. (**A**) Immunofluorescent analysis of pro-fibrotic markers (αSMA and COL1A1) on sectioned vCM-MTs following a 2 days treatment with TGFβ alone or in combination with a TGFβ-receptor I inhibitor (SB431542) or a BET bromodomain inhibitor (JQ1). The scale bar represents 100 µm. (**B**) Representative haematoxylin and eosin (H&E) and Picro Sirius Red stained images of vCM MTs treated with TGFβ alone or in combination with SB431542 or JQ1. Quantification of Picro Sirius Red is shown as a percentage of the total microtissue (right; *n* = 10 from 2 independent experiments). The scale bar represents 75 µm. (**C**) Gene expression analysis of pro-fibrotic genes (*ACTA2*, *COL1A1*, *COL1A2* and *POSTN*) in vCM MTs treated with TGFβ alone or in combination with SB431542 (SB) or JQ1. Data are presented as mean ± SD (*n* = 3 independent experiments) relative to control vCM-MTs. Statistical analysis was performed using a Kruskal-Wallis test. (**D,E**) Ca^2+^ dynamics in vCM MTs treated with TGFβ alone or in combination with SB431542 (SB) or JQ1. (**D**) Averaged Ca^2+^ traces of treated vCM MTs. Error bars represent SEM. (**E**) Quantification of Ca^2+^ time to peak (F_max_/F_0_), Ca^2+^ tau, Ca^2+^ transient duration 50% (CaTD50) and Ca^2+^ transient duration (CaTD). Data are presented as mean ± SD (*n* = 16 control, *n* = 15 TGFβ, *n* = 15 TGFβ + SB, *n* = 17 TGFβ + JQ1 treated vCM MTs from 4 independent experiments). Statistical analysis was performed using a Kruskal-Wallis test. (**F**) Gene expression analysis of calcium handling genes (*ATP2A2*, *RYR2* and *CACNA1C*) in vCM MTs treated with TGFβ alone or in combination with SB or JQ1. Data are presented as mean ± SD (*n* = 3 independent experiments) relative to control vCM-MTs. Statistical analysis was performed using a Kruskal-Wallis test. (**G**) Gene expression analysis of endothelial (*CDH5* and *PECAM1*) and cardiac (*TNNT2*, *ACTN2*, *DES* and *GJA1*) and (**H**) In vCM MTs treated with TGFβ alone or in combination with SB or JQ1. Data are presented as mean ± SD (*n* = 3 independent experiments) relative to control vCM-MTs. Statistical analysis was performed using a Kruskal-Wallis test.

Having shown the profibrotic remodelling in aCM MTs following TGFβ treatment, we next focused on the responses of vCM MTs to similar profibrotic stimulation. Like aCM MTs, TGFβ potently upregulated the expression of αSMA and COL1A1 in vCM MTs, as observed by immunofluorescence staining (Figure 4A). TGFβ treatment increased deposition of collagen, as shown by Picro Sirius red staining (Figure 4B). In addition, TGFβ treatment led to an upregulation in the expression of pro-fibrotic genes (*ACTA2*, *COL1A1*, *COL1A2* and *POSTN*) (Figure 4C). By adding either SB431542 or JQ1 inhibitors, we observed a reduction in αSMA and COL1A1 expression, as shown by immunofluorescence microscopy (Figure 4A), and a reduction in global collagen staining (Figure 4B). In addition, TGFβ plus SB431542 or JQ1 treatment restored the expression of *ACTA2*, *COL1A1*, *COL1A2* and *POSTN* to basal levels (Figure 4C), mimicking a similar response to what was observed in aCM MTs.

Similar to the phenotypes observed in aCM MTs, TGFβ treatment of vCM MTs resulted in a more prolonged Ca^2+^ trace (Figure 4D), that was associated with no changes in Ca^2+^ peak fluorescence, but revealed an increase in Ca^2+^ decay and a more prolonged Ca^2+^ transient (Figure 4D), indicative of dysregulated Ca^2+^ handling which correlated to cardiac dysfunction. We next assessed the effects of fibrosis blockade using SB431542 and JQ1 inhibitors. In vCM MTs, combined treatment of TGFβ and SB431542 shortened the prolonged Ca^2+^ trace that was observed with TGFβ treatment alone (Figure 4D). Interestingly, combined TGFβ treatment with SB431542 was not sufficient to reduce Ca^2+^ decay, even if significantly shortening the Ca^2+^ transient duration (Figure 4E). Similarly, combined treatment of TGFβ with JQ1 did not alter Ca^2+^ decay, but significantly shortened the overall Ca^2+^ transient duration (Figure 4E). Additional analysis of Ca^2+^ dynamics in vCM MTs revealed the presence of ectopic calcium sparks following TGFβ treatment (Supplementary Figure S5B) with a prolonged calcium time-to-peak (Supplementary Figure S5D). This wasn't accompanied by changes in beating rate. These phenotypes could be reversed in the presence of either SB431542 or JQ1 treatment. TGFβ treatment of vCM MTs showed reductions in the expression of *ATP2A2* and *RYR2*, which were restored following SB431542 or JQ1 treatment (Figure 4F). No differences in the expression of *CACNA1C* were observed following TGFβ treatment of vCM MTs alone or in combination with SB431542 or JQ1 (Figure 4F). TGFβ treatment of vCM MTs reduced *PECAM1* expression and downregulated cardiac gene expression too (Figures 4G,H). The combined treatment of TGFβ and SB431542 restored the expression levels of *PECAM1* (Figure 4G). In addition, *ACTN2*, *DES* and *GJA1*, but not *TNNT2*, gene expression levels returned to basal levels following the combined treatment of TGFβ and SB431542 (Figure 4H). Finally, the combined treatment of TGFβ and JQ1 in vCM MTs restored the fibrotic gene expression of *CDH5*, *PECAM1*, *TNNT2* and *DES* but not *ACTN2* or *GJA1* (Figures 4G,H).

Collectively, this data demonstrate the spatio-temporal interactions that occur between cardiomyocytes, fibroblasts and endothelial cells within atrial and ventricular microenvironments following pro-fibrotic activation and offer insights into plausible therapeutics to treat cardiac fibrosis.

## Discussion

As a leading cause of death worldwide, there is a pressing need for improved translational models for both the study of complex cardiac pathologies, but also the robust and scalable interrogation of new therapeutic interventions (6). The development of cardiac microtissues (also referred to as cardiac organoids and/or cardioids) offer a route towards addressing this need. Recent advances in hiPSC technologies have allowed the production of several cardiac microtissue models (23, 37, 38, 43). However, intrinsic limitation within the current cardiac microtissue models, such as variability in composition and structural organisation, and lack of patterned vasculature limit their application. Moreover, current cardiac models have largely been utilised to study cardiac developmental biology, with limited insights into the utilisation of these models to assess cardiac pathology (37, 38).

Here, we present a highly robust protocol for producing chamber-specific cardiac microtissues involving the simultaneous differentiation of vascular sprouts and atrial or ventricular cardiomyocytes from hiPSCs. When combined, these formed 3D cardiac microtissues that retained a patterned vascular network. By using a parallel differentiation methodology, our protocol controls for the number of cardiomyocytes that are added to form the 3D cardiac microtissue and recapitulate the physiological cell ratios seen in adult healthy cardiac tissue (51, 52). By combining cardiomyocytes with a vascular sprout grown in a 3D matrix, we promote the development of an integrated cardiac vascular network, offering a method to investigate cardiac cellular interactions in a microtissue environment which recapitulates the microvasculature of the human heart. These tend to rely on a single differentiation protocol to produce multiple cell types that form a patterned 3D structure but lack branched endothelial architecture (37, 43, 61). Moreover, several of the current models have relied on the use of fibroblasts and endothelial cells from dermal, foetal or venular origin, which, although human in nature, may differentially impact on cardiomyocyte behaviour and function within the microtissues (15, 27, 29, 31, 34). Although the existence of vascular endothelial cells within cardiac microtissue models has been described (23, 38, 43), the low abundance and lack of tubular structure within the microtissues preclude them from presenting the physiologically relevant communication with stromal and cardiac cell types (61, 62). Therefore, our cardiac microtissue models may provide a reliable platform for investigating chamber-specific pathologies associated with changes in interactions between endothelial cells, cardiomyocytes and stromal cells within the cardiac tissue niche.

Cardiac microtissues have been used to investigate various aspects of cardiac physiology and pathophysiology. Several recent studies have focused on the generation of cardiac microtissue models primarily composed of ventricular cardiomyocytes (23, 43). These models are derived from common differentiation protocols which involve self-aggregation of multiple cell types to form beating microtissues. Although integral to the study of ventricular cell biology, these models do not take into consideration the anatomical presence of the atria. More recently, studies have focused on the generation of atrial microtissues to understand atrial biology (37, 38). Consistent with these studies, our chamber-specific cardiac microtissues resemble many aspects of healthy atrial and ventricular cardiac tissue (14). In addition, by using optical mapping of aCM MTs and vCM MTs we were able to show that atrial and ventricular Ca^2+^ dynamics are unique, with aCM MTs having shortened Ca^2+^ transients and a quicker Ca^2+^ decay compared to vCM MTs, which is consistent with 2D cultured atrial and ventricular cardiomyocytes derived from hiPSCs (49).

Two of the ultimate goals for generating hiPSC-derived cardiac microtissues is to model human disease and create a reliable platform for drug discovery. With this in mind, we focused on recapitulating cardiac fibrosis, a pathological complication observed in heart failure patients. Previous studies have demonstrated that cardiac fibrosis can be induced in cardiac microtissues (18, 63). Consistent with these studies, our vCM-MTs demonstrated hallmarks of fibrosis in the presence of TGFβ, with evidence of fibroblast to myofibroblast activation (elevations in αSMA staining) and excessive deposition of collagen.

Similarly, our study showed aCM MTs react to TGFβ in a similar manner to vCM MTs. Cardiomyocyte cell death is a hallmark observation following persistent TGFβ exposure in conditions of heart failure (65). In our model, exposure of TGFβ reduced the expression of cardiac and endothelial markers in vCM MTs. Importantly, this phenotype was seen to a lesser extent in aCM MTs, suggesting divergent mechanisms of fibrosis atrium vs. ventricle. Functionally, induction of fibrosis altered Ca^2+^ dynamics in both aCM MTs and vCM MTs and resulted in a more prolonged Ca^2+^ transient, contrary to a previous report that showed shortening in Ca^2+^ transients with increased Ca^2+^ sparks in cardiac microtissues subjected to hypoxia-induced cardiac fibrosis (18). The differences in Ca^2+^ dynamics observed between the two models are likely due to the markedly different methods applied for the induction of cardiac fibrosis (hypoxia in the Richards et al. study vs. TGFβ-induced in our study), the nature of the model used, and the post analysis (regions of interest in the Richards et al. study vs. whole microtissue in our study). It remains to be shown if cardiomyocyte cell death within the cardiac microtissues accounts for the divergent effects observed following fibrosis induction.

When looking to block the effects of TGFβ treatment, both TGFβ receptor I inhibition (SB431542) and BET bromodomain inhibition (JQ1) were able to prevent the TGFβ-induced pro-fibrotic effects observed in vCM MTs. In aCM MTs, only JQ1 treatment could reverse the pro-fibrotic effects induced by TGFβ, but not SB431542. One plausible reason for the lack of fibrosis blockade seen in aCM MTs treated with SB431542 could be due to the fact that TGFβ can exert its effects via non-canonical signalling pathways (46). Given the preferential targeting of SB431542 to the Activin receptor like kinase-5 (TGFβ receptor I) protein (55), it remains to be shown if differential TGFβ receptor 1 expression levels between the aCM MTs and vCM MTs could explain the differences in the results observed. BET bromodomain inhibition has been identified as a method to block cardiac fibrosis (18, 57, 58, 66). More recently, the mechanisms of JQ1's action have been explored in the context of cardiac fibrosis with the identification of the transcriptional factor *Meox1* as a central target that BET promotes transcription of via an enhancer/promoter interaction (56, 57). It remains to be shown if similar mechanisms are required in JQ1-medicated fibrosis blockade in our cardiac microtissue models.

In addition to these common findings between aCM MTs and vCM MTs, there were several findings that were unique to the chambered microtissues. When looking at Ca^2+^ dynamics in the microtissues, calcium decay was reduced following combined treatment of aCM MTs with TGFβ and JQ1 when compared to TGFβ treated aCM MTs. This observation was not observed in vCM MTs most likely due to the higher degree of variability in calcium decay dynamics observed. Interestingly, at the molecular level, vCM MTs showed reductions in key Ca^2+^ handling genes following TGFβ treatment which could potentially be due to direct electrical remodelling in the presence of TGFβ that has previously been shown to reduce RyR2 and SERCA function in neonatal rat ventricular cardiomyocytes (67, 68). As to why this observation was more pronounce in vCM MTs compared to aCM MTs remains to be determined.

We show the importance of chamber specific cardiac microtissues in the context of dissecting specific effects of remodelling. Importantly, by assessing the impact of treatments on gene expression, microtissue architecture, and function through calcium dynamics, we demonstrate the need for multi-parametric assessment of the effects of both fibrosis and potential pharmacological interventions. Ultimately, for anti-fibrotic agents to be effective in the treatment of cardiac pathologies, a reversal or restoration of function is required, and our proof of principle human cardiac microtissue models offer insights into reversing cardiac fibrosis, e.g., through the use of JQ1. Our platform is highly scalable, reproducible, and will robustly allow for the assessment of cardiac pathologies including, but not limited to, cardiac fibrosis.

### Limitations of study

Compared to primary adult human atrial and ventricular cardiac tissues, there are still fundamental differences in the electrophysiological maturation of aCM MTs and vCM MTs; however, these microtissues do show improved maturation over their 2D counterparts. To robustly demonstrate improved maturation, investigating improved alignment of sarcomeres (through imaging of cTnT and α-actinin) and a quantitative comparison between 2D and 3D cultures reported herein is needed. This is an important avenue of future research, and will require the optimisation of high resolution, volumetric imaging of microtissues. While a previously published approach (23) whereby microtissues are disaggregated and replated to allow for a sample which is optically amenable for high resolution imaging can be used (Supplementary Figure S6), an ideal experiment would image a thick section of microtissue to establish sarcomeric alignment in 3D. Future work will focus on establishing methods to robustly assess maturation using sarcomere alignment, and leverage that approach to use microtissues as a means by which to assess pathological perturbations underlying various cardiomyopathies. Similarly, further biochemical and proteomic/transcriptomic characterisation over time will be critical to understand the true effects of maturation within the 3D microtissue environment. In addition, although the generation of aCM MTs and vCM MTs is highly reproducible based on morphology, gene and protein profiling, the inter- and/or intra-batch microtissue variability does affect the microtissue response to pro-fibrotic agents. For example, when looking at Ca^2+^ dynamics in the cardiac microtissues, variability in dye loading altered the Ca^2+^ signal per microtissue. This could be minimised using an hiPSC line that contains a Ca^2+^ reporter dye that have previously been developed (38, 69). The 3D vascularised endothelial structure observed in the microtissues is an advance on current cardiac microtissue models but it remains to be shown if these structures are perfusable.

## Experimental procedures

### Resource availability

#### Materials availability

Complete step-by-step protocols are available on request.

#### Code availability

No code was generated or used in this manuscript.

#### Induction of vascular sprouts from human pluripotent stem cells (hiPSCs)

The commercially available human hiPSC line (Gibco) was used in this study. The hiPSCs were maintained in an undifferentiated state by culturing with feeder-free conditions using StemFlex medium (Thermo Fisher) on plates coated with Geltrex (Thermo Fisher) at 37°C in 5% CO_2_ with 95% air. hiPSCs were differentiated into endothelial sprouts using a previously described method with slight modifications (47, 48). Undifferentiated hiPSCs cultured on Geltrex were dissociated using the EDTA passaging method and incubated overnight in StemFlex supplemented with RevitaCell (Thermo Fisher Scientific). On the following day the resulting aggregates were collected via centrifugation at 300x*g* for 5 min and resuspended in StemPro differentiation media (Thermo Fisher), supplemented with 6 µm CHIR99021 (MilliporeSigma) and 50 ng/ml VEGFA-165 Vascular Endothelial Growth Factor-A (VEGF-165, StemCell Technologies), Fibroblast Growth Factor-2 (FGF2, StemCell Technologies), and Bone Morphogenic Protein-4 (BMP4, Thermo Fisher). Cells were grown for 3 days before collection via gravitation and resuspended in fresh StemPro supplemented with 50 ng/ml VEGFA and FGF2, as well as 2 µm Forskolin (Sigma). At day 5 of the protocol, aggregates were collected once more via gravitation before hydrogel embedding (48).

Hydrogel embedding was performed as follows: Aliquots of Matrigel (Corning) were allowed to thaw on ice and mixed with VitroCol Human Collagen Type I (Advanced Biomatrix) at a 30:70 ratio. 400 µl base layers of this mixture were neutralized with 1M NaOH and added to pre-wetted wells of a 12-well plate. The base layer was allowed to polymerise for 90 min within a cell culture incubator before a second 300 µl layer containing cell aggregates was prepared and added. This was allowed to polymerize for a further 90 min, before 1 ml/well of StemPro supplemented with 15% FBS (Thermo Fisher), 5U/ml Heparin, and 100 ng VEGFA and FGF2 was added. Fresh media was added at day 7, and sprouts were collected on day 10 by trituration of hydrogels and centrifugation, before individual sprouts were added to a 96 well ultra-low attachment plate.

#### Generation of atrial and ventricular cardiomyocytes from hiPSCs

Atrial and ventricular cardiomyocytes were generated through the modulation of Wnt signalling using a previously published method (49). Colonies of hiPSCs were detached using Tryple (Thermo Fisher), and 200,000 cells were plated on Geltrex coated tissue culture plates (Thermo Fisher). Once 90% confluent, the media was changed to RPMI 1640 (Thermo Fisher) supplemented with 0.2 mg/ml L-ascorbic acid (Sigma-Aldrich) and 4 µm CHIR99021 (Sigma-Aldrich) to promote mesoderm differentiation. 48 h after, the medium was replaced with RPMI 1640 and L-ascorbic acid containing 5 µm IWP2 (Sigma-Aldrich) to promote cardiac progenitor differentiation. The cells were further cultured in basal media for an additional 96 h in RPMI 1640 with L-ascorbic acid, after which the media was changed to RPMI 1640 containing 2% B27 supplement (Thermo Fisher). To specify atrial cardiomyocytes, retinoic acid (Sigma-Aldrich) was added to the media for 72 h after day 3 of differentiation until day 6. Beating cultures were observed between day 8 and 10.

#### Cardiac microtissue formation

To generate 3D atrial (aCM MTs) and ventricular (vCM MTs) cardiac microtissues, day 20 atrial or ventricular cardiomyocytes were dissociated into single cells using Accutase (Thermo Fisher), and 5,000 cells (per well) were aggregated with individual vascular sprouts in StemPro medium (Life Technologies) supplemented with 2% B27 and 5% penicillin/streptomycin using ultra-low attachment 96-well plates (Corning). The plate was gently centrifuged at 300 rpm for 3 min to promote cardiomyocyte clumping and maintained at 37°C in 5% CO_2_ with 95% air with media being replaced every 2 days.

For experiments involving chemical treatments, cardiac microtissues were incubated with 25 ng/ml transforming growth factor-β (TGFβ) for 48 h. For studies involving inhibitors, similar 48-h incubations were performed with recombinant TGFβ (25 ng/ml) the TGFβ receptor I inhibitor, SB431542, was used at 25 um and the BET bromodomain inhibitor, JQ1, was used at 10 nm. Inhibitors were added simultaneously with chemical treatments.

#### Flow cytometry

A minimum of 12 aCM MTs and vCM MTs were collected per experiment and allowed to sediment by gravity before being washed once with PBS (Sigma-Aldrich) and subjected to flow cytometry processing as previously described (70). Briefly, aCM MTs and vCM MTs were dissociated to single cells with the treatment of Collagenase Type II (Worthington Biochemical) in HBSS (Sigma-Aldrich) for 10 min at 37°C. Single cells were centrifuged at 300x*g* followed by two washes with PBS before being blocked for 30 min in blocking buffer (PBS supplemented with 2% FBS and 2% BSA). The single cells were stained using the following antibodies: FITC-conjugated cardiac troponin-T (cTnT) (Miltenyi Biotec), PE-conjugated platelet derived growth factor receptor-beta (PDGFRβ) (BioLegend), APC-Cy7 conjugated vascular endothelial-cadherin (CD144) (BioLegend), PE-Cy7 conjugated Platelet-endothelial cell adhesion molecule (CD31) (BioLegend) and APC conjugated neural/glial antigen 2 (NG2) (BioLegend). Samples were processed on the BD LSR Fortesa using FACSDiva software (BD Biosciences) followed by offline analysis using FlowJo (FlowJo, LCC).

#### RNA isolation and RT-qPCR

RNA was isolated by combining a minimum of 12 aCM-MTs or vCM-MTs per experiment using the RNeasy mini kit (QIAGEN). Reverse transcription was carried out using the High-Capacity cDNA synthesis kit (Thermo Fisher) according to the manufacturer's protocol. qPCR was carried out using PowerUP SYBR green master mix (Applied Biosystems) on a QuantStudio 3 Real-Time PCR system (Thermo Fisher). All primers were obtained from the Integrated DNA Technologies PrimeTime qPCR Predesigned Primer Library (https://eu.idtdna.com/pages/products/qpcr-and-pcr/gene-expression/primetime-primer-only-assays) (see Supplementary Table S1). All results were normalised using *GAPDH* as the housekeeping gene.

#### Immunofluorescent imaging

A minimum of 6 aCM MTs and vCM MTs per experiment were collected into 15 ml falcon tubes and allowed to sediment by gravitation before being fixed in 4% paraformaldehyde (PFA). Samples were then washed in 0.05% PBS-Tween (PBST) and incubated in blocking buffer (2% goat serum, 1% BSA, 1% Triton X-100 and sodium deoxycholate) overnight. Samples were then incubated with primary antibodies (see Supplementary Table S2) for an additional 24 h at 4°C under mild agitation, after which samples were incubated with Alexa Fluor secondary antibodies (see Supplementary Table S2) for 2 h at room temperature. The samples were then washed before being labelled with 4′6-diamidino-2-phenylindole (DAPI) and washed an additional time using PBST. To prevent samples from moving during acquisition, samples were mounted using 0.5% ultra-low melting point agarose (Fisher Scientifc) in Ibidi 8-well slides (Ibidi), followed by serial dehydration in ethanol (30%, 50%, 70% and 100%), clearing in ethyl cinnamate and imaging using a Zeiss LSM 880 Airyscan confocal microscope. IMARIS was used to generate 3D-rendered images.

For sarcomere alignment experiments, aCM MTs and vCM MTs were dissociated with collagenase Type II (*as explained above for Flow Cytometry*) and replated in Ibidi 8-well slides prior to staining with α-actinin. Images were acquired on a Zeiss LSM 880 Airyscan confocal microscope.

#### Immunohistochemistry

5 aCM MTs and vCM MTs per experiment were fixed for 30 min in 4% PFA at room temperature. For paraffin embedded sections, samples underwent serial dehydration using 30%, 50%, 70%, 90%, 100% ethanol and finally Histo-clear II (Scientific Laboratory Supplies). Samples were embedded in paraffin and 4 µm sections were collected using the HistoCore AUOTOCUT microtome (Leica Biosystems). H&E and Picro Sirius Red staining was performed as per standard protocols by Advanced Histopathology Laboratory LTD. Quantification of Picro Sirius Red staining was carried out as previously described (71). For immunostaining, sections were de-paraffinized and subjected to antigen retrieval procedure in citrate buffer (pH 6.0, 95°C) for 5 min before staining. For frozen sections, fixed samples were embedded using Tissue-Tek O.C.T. Compound (Sakura Finetek) as previously described (70) and 8 µm sections were collected using a cryostat (Leica Biosystems).

#### Ca^2+^ imaging in cardiac microtissues by optical mapping

6 aCM MTs and vCM MTs per experiment were allowed to sediment by gravity and loaded with 8 µm Rhod-2 AM fluorescent calcium indicator (Thermo Fisher) and 0.02% Pluoronic F-127 (Thermo Fisher) in Tyrode's solution containing 140 mm NaCl, 5.4 mm KCl, 1.8 mm CaCl_2_, 1 mm MgCl_2_, 10 mm HEPES and 10 mm glucose (pH 7.4) for 20 min at room temperature. Cardiac microtissues were subsequently washed with Tyrode's solution before being acquired on a Zeiss Epi fluorescent microscope. Post-acquisition analysis was carried out using Caltrack, a MatLab plugin for intracellular Ca^2+^ analysis (69).

#### Quantification and statistical analysis

Statistical analyses were performed using GraphPad Prism 6.0 (GraphPad Software, Inc., CA, USA), using unpaired two-tailed Student *t*-tests. Statistical analysis for Ca^2+^ transient measurements were performed using non-parametric Kruskal-Wallis with a *post hoc* Dunn-Holland-Wolf test. For comparisons between multiple groups, a Two-way ANOVA followed by a Bonferroni *post-hoc* comparison test was used, unless noted otherwise. Statistically significant *P*-values (<0.05) were noted on graphs. The total number of replicates (*n*) are noted in the figure legends.

## Data Availability

The raw data supporting the conclusions of this article will be made available by the authors, without undue reservation.

## References

[1] Diseases GBD, Injuries C. Global burden of 369 diseases and injuries in 204 countries and territories, 1990–2019: a systematic analysis for the global burden of disease study 2019. Lancet. (2020) 396(10258):1204–22. 10.1016/S0140-6736(20)30925-9 33069326 PMC7567026

[2] Mc Namara K Alzubaidi H Jackson JK. Cardiovascular disease as a leading cause of death: how are pharmacists getting involved? Integr Pharm Res Pract. (2019) 8:1–11. 10.2147/IPRP.S133088 30788283 PMC6366352

[3] Roth GA Mensah GA Johnson CO Addolorato G Ammirati E Baddour LM Global burden of cardiovascular diseases and risk factors, 1990–2019: update from the GBD 2019 study. J Am Coll Cardiol. (2020) 76(25):2982–3021. 10.1016/j.jacc.2020.11.010 33309175 PMC7755038

[4] Khakoo AY Yurgin NR Eisenberg PR Fonarow GC. Overcoming barriers to development of novel therapies for cardiovascular disease: insights from the oncology drug development experience. JACC Basic Transl Sci. (2019) 4(2):269–74. 10.1016/j.jacbts.2019.01.011 31061928 PMC6488739

[5] Cho S Lee C Skylar-Scott MA Heilshorn SC Wu JC. Reconstructing the heart using iPSCs: engineering strategies and applications. J Mol Cell Cardiol. (2021) 157:56–65. 10.1016/j.yjmcc.2021.04.006 33895197 PMC8378256

[6] van der Velden J Asselbergs FW Bakkers J Batkai S Bertrand L Bezzina CR Animal models and animal-free innovations for cardiovascular research: current status and routes to be explored. Consensus document of the ESC working group on myocardial function and the ESC working group on cellular biology of the heart. Cardiovasc Res. (2022) 9:3016–51. 10.1093/cvr/cvab370 PMC9732557 34999816

[7] Lian X Hsiao C Wilson G Zhu K Hazeltine LB Azarin SM Robust cardiomyocyte differentiation from human pluripotent stem cells via temporal modulation of canonical Wnt signaling. Proc Natl Acad Sci U S A. (2012) 109(27):E1848–57. 10.1073/pnas.1200250109 22645348 PMC3390875

[8] Lian X Zhang J Azarin SM Zhu K Hazeltine LB Bao X Directed cardiomyocyte differentiation from human pluripotent stem cells by modulating Wnt/beta-catenin signaling under fully defined conditions. Nat Protoc. (2013) 8(1):162–75. 10.1038/nprot.2012.150 23257984 PMC3612968

[9] Burridge PW Matsa E Shukla P Lin ZC Churko JM Ebert AD Chemically defined generation of human cardiomyocytes. Nat Methods. (2014) 11(8):855–60. 10.1038/nmeth.2999 24930130 PMC4169698

[10] Sharma A Li G Rajarajan K Hamaguchi R Burridge PW Wu SM. Derivation of highly purified cardiomyocytes from human induced pluripotent stem cells using small molecule-modulated differentiation and subsequent glucose starvation. J Vis Exp. (2015) 97:52628. 10.3791/52628. PMC4401368 25867738

[11] Karbassi E Fenix A Marchiano S Muraoka N Nakamura K Yang X Cardiomyocyte maturation: advances in knowledge and implications for regenerative medicine. Nat Rev Cardiol. (2020) 17(6):341–59. 10.1038/s41569-019-0331-x 32015528 PMC7239749

[12] Campostrini G Windt LM van Meer BJ Bellin M Mummery CL. Cardiac tissues from stem cells: new routes to maturation and cardiac regeneration. Circ Res. (2021) 128(6):775–801. 10.1161/CIRCRESAHA.121.318183 33734815 PMC8410091

[13] Mohr E Thum T Bar C. Accelerating cardiovascular research: recent advances in translational 2D and 3D heart models. Eur J Heart Fail. (2022) 24:1778–91. 10.1002/ejhf.2631 35867781

[14] Thomas D Choi S Alamana C Parker KK Wu JC. Cellular and engineered organoids for cardiovascular models. Circ Res. (2022) 130(12):1780–802. 10.1161/CIRCRESAHA.122.320305 35679369 PMC12034489

[15] Richards DJ Coyle RC Tan Y Jia J Wong K Toomer K Inspiration from heart development: biomimetic development of functional human cardiac organoids. Biomaterials. (2017) 142:112–23. 10.1016/j.biomaterials.2017.07.021 28732246 PMC5562398

[16] Drakhlis L Biswanath S Farr CM Lupanow V Teske J Ritzenhoff K Human heart-forming organoids recapitulate early heart and foregut development. Nat Biotechnol. (2021) 39(6):737–46. 10.1038/s41587-021-00815-9 33558697 PMC8192303

[17] Lee J Sutani A Kaneko R Takeuchi J Sasano T Kohda T In vitro generation of functional murine heart organoids via FGF4 and extracellular matrix. Nat Commun. (2020) 11(1):4283. 10.1038/s41467-020-18031-5 32883967 PMC7471119

[18] Richards DJ Li Y Kerr CM Yao J Beeson GC Coyle RC Human cardiac organoids for the modelling of myocardial infarction and drug cardiotoxicity. Nat Biomed Eng. (2020) 4(4):446–62. 10.1038/s41551-020-0539-4 32284552 PMC7422941

[19] Kupfer ME Lin WH Ravikumar V Qiu K Wang L Gao L In situ expansion, differentiation, and electromechanical coupling of human cardiac muscle in a 3D bioprinted, chambered organoid. Circ Res. 2020;127(2):207–24. 10.1161/CIRCRESAHA.119.316155 32228120 PMC8210857

[20] Sasaki D Matsuura K Seta H Haraguchi Y Okano T Shimizu T. Contractile force measurement of human induced pluripotent stem cell-derived cardiac cell sheet-tissue. PloS One. (2018) 13(5):e0198026. 10.1007/978-1-0716-1484-6_16 29791489 PMC5965888

[21] Jabbour RJ Owen TJ Pandey P Reinsch M Wang B King O In vivo grafting of large engineered heart tissue patches for cardiac repair. JCI Insight. (2021) 6(15):e144068. 10.1172/jci.insight.144068 34369384 PMC8410032

[22] Noguchi R Nakayama K Itoh M Kamohara K Furukawa K Oyama JI Development of a three-dimensional pre-vascularized scaffold-free contractile cardiac patch for treating heart disease. J Heart Lung Transplant. (2016) 35(1):137–45. 10.1016/j.healun.2015.06.001 26433566

[23] Giacomelli E Meraviglia V Campostrini G Cochrane A Cao X van Helden RWJ Human-iPSC-derived cardiac stromal cells enhance maturation in 3D cardiac microtissues and reveal non-cardiomyocyte contributions to heart disease. Cell Stem Cell. (2020) 26(6):862–79.e11. 10.1016/j.stem.2020.05.004 32459996 PMC7284308

[24] Giacomelli E Mummery CL Bellin M. Human heart disease: lessons from human pluripotent stem cell-derived cardiomyocytes. Cell Mol Life Sci. (2017) 74(20):3711–39. 10.1007/s00018-017-2546-5 28573431 PMC5597692

[25] Kensah G Roa Lara A Dahlmann J Zweigerdt R Schwanke K Hegermann J Murine and human pluripotent stem cell-derived cardiac bodies form contractile myocardial tissue in vitro. Eur Heart J. (2013) 34(15):1134–46. 10.1093/eurheartj/ehs349 23103664

[26] Rossi G Broguiere N Miyamoto M Boni A Guiet R Girgin M Capturing cardiogenesis in gastruloids. Cell Stem Cell. (2021) 28(2):230–40.e6. 10.1016/j.stem.2020.10.013 33176168 PMC7867643

[27] Stevens KR Kreutziger KL Dupras SK Korte FS Regnier M Muskheli V Physiological function and transplantation of scaffold-free and vascularized human cardiac muscle tissue. Proc Natl Acad Sci U S A. (2009) 106(39):16568–73. 10.1073/pnas.0908381106 19805339 PMC2746126

[28] Stevens KR Pabon L Muskheli V Murry CE. Scaffold-free human cardiac tissue patch created from embryonic stem cells. Tissue Eng Part A. (2009) 15(6):1211–22. 10.1089/ten.tea.2008.0151 19063661 PMC2774496

[29] Ravenscroft SM Pointon A Williams AW Cross MJ Sidaway JE. Cardiac non-myocyte cells show enhanced pharmacological function suggestive of Contractile maturity in stem cell derived cardiomyocyte microtissues. Toxicol Sci. (2016) 152(1):99–112. 10.1093/toxsci/kfw069 27125969 PMC4922542

[30] Giacomelli E Bellin M Sala L van Meer BJ Tertoolen LG Orlova VV Three-dimensional cardiac microtissues composed of cardiomyocytes and endothelial cells co-differentiated from human pluripotent stem cells. Development. (2017) 144(6):1008–17. 10.1242/dev.143438 28279973 PMC5358113

[31] Pointon A Pilling J Dorval T Wang Y Archer C Pollard C. From the cover: high-throughput imaging of cardiac microtissues for the assessment of cardiac contraction during drug discovery. Toxicol Sci. (2017) 155(2):444–57. 10.1093/toxsci/kfw227 28069985

[32] Devarasetty M Forsythe S Shupe TD Soker S Bishop CE Atala A Optical tracking and digital quantification of beating behavior in bioengineered human cardiac organoids. Biosensors. (2017) 7(3):24. 10.3390/bios7030024 28644395 PMC5618030

[33] Correia C Koshkin A Duarte P Hu D Carido M Sebastiao MJ 3D aggregate culture improves metabolic maturation of human pluripotent stem cell derived cardiomyocytes. Biotechnol Bioeng. (2018) 115(3):630–44. 10.1002/bit.26504 29178315

[34] Beauchamp P Jackson CB Ozhathil LC Agarkova I Galindo CL Sawyer DB 3D co-culture of hiPSC-derived cardiomyocytes with cardiac fibroblasts improves tissue-like features of cardiac spheroids. Front Mol Biosci. (2020) 7:14. 10.3389/fmolb.2020.00014 32118040 PMC7033479

[35] Varzideh F Pahlavan S Ansari H Halvaei M Kostin S Feiz MS Human cardiomyocytes undergo enhanced maturation in embryonic stem cell-derived organoid transplants. Biomaterials. (2019) 192:537–50. 10.1016/j.biomaterials.2018.11.033 30529872

[36] Filippo Buono M von Boehmer L Strang J Hoerstrup SP Emmert MY Nugraha B. Human cardiac organoids for modeling genetic cardiomyopathy. Cells. (2020) 9(7):1733. 10.3390/cells9071733 32698471 PMC7409052

[37] Hofbauer P Jahnel SM Papai N Giesshammer M Deyett A Schmidt C Cardioids reveal self-organizing principles of human cardiogenesis. Cell. (2021) 184(12):3299–317.e22. 10.1016/j.cell.2021.04.034 34019794

[38] Silva AC Matthys OB Joy DA Kauss MA Natarajan V Lai MH Co-emergence of cardiac and gut tissues promotes cardiomyocyte maturation within human iPSC-derived organoids. Cell Stem Cell. (2021) 28(12):2137–52.e6. 10.1016/j.stem.2021.11.007 34861147

[39] Ng WH Johnston EK Tan JJ Bliley JM Feinberg AW Stolz DB Recapitulating human cardio-pulmonary co-development using simultaneous multilineage differentiation of pluripotent stem cells. Elife. (2022) 11:e67872. 10.7554/eLife.67872. PMC8846595 35018887

[40] Andersen P Tampakakis E Jimenez DV Kannan S Miyamoto M Shin HK Precardiac organoids form two heart fields via Bmp/Wnt signaling. Nat Commun. (2018) 9(1):3140. 10.1038/s41467-018-05604-8 30087351 PMC6081372

[41] Lewis-Israeli YR Wasserman AH Gabalski MA Volmert BD Ming Y Ball KA Self-assembling human heart organoids for the modeling of cardiac development and congenital heart disease. Nat Commun. (2021) 12(1):5142. 10.1038/s41467-021-25329-5 34446706 PMC8390749

[42] Mourad O Yee R Li M Nunes SS. Modeling heart diseases on a chip: advantages and future opportunities. Circ Res. (2023) 132(4):483–97. 10.1161/CIRCRESAHA.122.321670 36795846

[43] Lewis-Israeli YR Volmert BD Gabalski MA Huang AR Aguirre A. Generating self-assembling human heart organoids derived from pluripotent stem cells. J Vis Exp. (2021) 175:63097. 10.3791/63097. PMC8496916 34605811

[44] Polonchuk L Chabria M Badi L Hoflack JC Figtree G Davies MJ Cardiac spheroids as promising in vitro models to study the human heart microenvironment. Sci Rep. (2017) 7(1):7005. 10.1038/s41598-017-06385-8 28765558 PMC5539326

[45] Zhang S Wan Z Kamm RD. Vascularized organoids on a chip: strategies for engineering organoids with functional vasculature. Lab Chip. (2021) 21(3):473–88. 10.1039/D0LC01186J 33480945 PMC8283929

[46] Frangogiannis NG. Transforming growth factor-beta in myocardial disease. Nat Rev Cardiol. (2022) 19(7):435–55. 10.1038/s41569-021-00646-w 34983937

[47] Wimmer RA Leopoldi A Aichinger M Kerjaschki D Penninger JM. Generation of blood vessel organoids from human pluripotent stem cells. Nat Protoc. (2019) 14(11):3082–100. 10.1038/s41596-019-0213-z 31554955

[48] Wimmer RA Leopoldi A Aichinger M Wick N Hantusch B Novatchkova M Human blood vessel organoids as a model of diabetic vasculopathy. Nature. (2019) 565(7740):505–10. 10.1038/s41586-018-0858-8 30651639 PMC7116578

[49] Cyganek L Tiburcy M Sekeres K Gerstenberg K Bohnenberger H Lenz C Deep phenotyping of human induced pluripotent stem cell-derived atrial and ventricular cardiomyocytes. JCI Insight. (2018) 3(12):e99941. 10.1172/jci.insight.99941 29925689 PMC6124434

[50] Devalla HD Schwach V Ford JW Milnes JT El-Haou S Jackson C Atrial-like cardiomyocytes from human pluripotent stem cells are a robust preclinical model for assessing atrial-selective pharmacology. EMBO Mol Med. (2015) 7(4):394–410. 10.15252/emmm.201404757 25700171 PMC4403042

[51] Tucker NR Chaffin M Fleming SJ Hall AW Parsons VA Bedi KC Jr Transcriptional and cellular diversity of the human heart. Circulation. (2020) 142(5):466–82. 10.1161/CIRCULATIONAHA.119.045401 32403949 PMC7666104

[52] Litvinukova M Talavera-Lopez C Maatz H Reichart D Worth CL Lindberg EL Cells of the adult human heart. Nature. (2020) 588(7838):466–72. 10.1038/s41586-020-2797-4 32971526 PMC7681775

[53] Nerbonne JM Kass RS. Molecular physiology of cardiac repolarization. Physiol Rev. (2005) 85(4):1205–53. 10.1152/physrev.00002.2005 16183911

[54] Henderson NC Rieder F Wynn TA. Fibrosis: from mechanisms to medicines. Nature. (2020) 587(7835):555–66. 10.1038/s41586-020-2938-9 33239795 PMC8034822

[55] Inman GJ Nicolas FJ Callahan JF Harling JD Gaster LM Reith AD SB-431542 is a potent and specific inhibitor of transforming growth factor-beta superfamily type I activin receptor-like kinase (ALK) receptors ALK4, ALK5, and ALK7. Mol Pharmacol. (2002) 62(1):65–74. 10.1124/mol.62.1.65 12065756

[56] Alexanian M Przytycki PF Micheletti R Padmanabhan A Ye L Travers JG A transcriptional switch governs fibroblast activation in heart disease. Nature. (2021) 595(7867):438–43. 10.1038/s41586-021-03674-1 34163071 PMC8341289

[57] Duan Q McMahon S Anand P Shah H Thomas S Salunga HT BET bromodomain inhibition suppresses innate inflammatory and profibrotic transcriptional networks in heart failure. Sci Transl Med. (2017) 9(390). 10.1126/scitranslmed.aah5084 28515341 PMC5544253

[58] Antolic A Wakimoto H Jiao Z Gorham JM DePalma SR Lemieux ME BET bromodomain proteins regulate transcriptional reprogramming in genetic dilated cardiomyopathy. JCI Insight. (2020) 5(15). 10.1172/jci.insight.138687 32603312 PMC7455078

[59] Mora MT Ferrero JM Gomez JF Sobie EA Trenor B. Ca(2+) cycling impairment in heart failure is exacerbated by fibrosis: insights gained from mechanistic simulations. Front Physiol. (2018) 9:1194. 10.3389/fphys.2018.01194 30190684 PMC6116328

[60] Feng J Armillei MK Yu AS Liang BT Runnels LW Yue L. Ca(2+) signaling in cardiac fibroblasts and fibrosis-associated heart diseases. J Cardiovasc Dev Dis. (2019) 6(4):34. 10.3390/jcdd6040034. 31547577 PMC6956282

[61] Feng W Schriever H Jiang S Bais A Wu H Kostka D Computational profiling of hiPSC-derived heart organoids reveals chamber defects associated with NKX2-5 deficiency. Commun Biol. (2022) 5(1):399. 10.1038/s42003-022-03346-4 35488063 PMC9054831

[62] Talman V Kivela R. Cardiomyocyte-Endothelial cell interactions in cardiac remodeling and regeneration. Front Cardiovasc Med. (2018) 5:101. 10.3389/fcvm.2018.00101 30175102 PMC6108380

[63] Flores-Vergara R Olmedo I Aranguiz P Riquelme JA Vivar R Pedrozo Z. Communication between cardiomyocytes and fibroblasts during cardiac ischemia/reperfusion and remodeling: roles of TGF-beta, CTGF, the renin angiotensin axis, and non-coding RNA molecules. Front Physiol. (2021) 12:716721. 10.3389/fphys.2021.716721 34539441 PMC8446518

[64] Lee MO Jung KB Jo SJ Hyun SA Moon KS Seo JW Modelling cardiac fibrosis using three-dimensional cardiac microtissues derived from human embryonic stem cells. J Biol Eng. (2019) 13:15. 10.1186/s13036-019-0139-6 30809271 PMC6375184

[65] Sweeney M Corden B Cook SA. Targeting cardiac fibrosis in heart failure with preserved ejection fraction: mirage or miracle? EMBO Mol Med. (2020) 12(10):e10865. 10.15252/emmm.201910865 32955172 PMC7539225

[66] Stratton MS Bagchi RA Felisbino MB Hirsch RA Smith HE Riching AS Dynamic chromatin targeting of BRD4 stimulates cardiac fibroblast activation. Circ Res. (2019) 125(7):662–77. 10.1161/CIRCRESAHA.119.315125 31409188 PMC7310347

[67] Ding Z Yuan J Liang Y Wu J Gong H Ye Y Ryanodine receptor type 2 plays a role in the development of cardiac fibrosis under mechanical stretch through TGFbeta-1. Int Heart J. (2017) 58(6):957–61. 10.1536/ihj.16-572 29162778

[68] Li S Li X Zheng H Xie B Bidasee KR Rozanski GJ. Pro-oxidant effect of transforming growth factor- beta1 mediates contractile dysfunction in rat ventricular myocytes. Cardiovasc Res. (2008) 77(1):107–17. 10.1093/cvr/cvm022 18006470

[69] Psaras Y Margara F Cicconet M Sparrow AJ Repetti GG Schmid M Caltrack: high-throughput automated calcium transient analysis in cardiomyocytes. Circ Res. (2021) 129(2):326–41. 10.1161/CIRCRESAHA.121.318868 34018815 PMC8260473

[70] Khan AO Reyat JS Hill H Bourne JH Colicchia M Newby ML Preferential uptake of SARS-CoV-2 by pericytes potentiates vascular damage and permeability in an organoid model of the microvasculature. Cardiovasc Res. (2022) 118:3085–96. 10.1093/cvr/cvac097 35709328 PMC9214165

[71] Courtoy GE Leclercq I Froidure A Schiano G Morelle J Devuyst O Digital image analysis of picrosirius red staining: a robust method for multi-organ fibrosis quantification and characterization. Biomolecules. (2020) 10(11):1585. 10.3390/biom10111585 33266431 PMC7709042

