# Supplementary material for: Modelling the pathology and treatment of cardiac fibrosis in vascularised atrial and ventricular cardiac microtissues
Source: Front Cardiovasc Med. 2023 Sep 1;10:1156759. doi: 10.3389/fcvm.2023.1156759 (PMC10506403; doi:10.3389/fcvm.2023.1156759)
Supplement: Supplementary file 1 [file Datasheet1.docx]

**Modelling the pathology and treatment of cardiac fibrosis in vascularised atrial and ventricular cardiac microtissues**

**Short title: Vascularised cardiac microtissues to study cardiac fibrosis**

Jasmeet S. Reyat *^1,2^, Alessandro di Maio ^3^, Beata Grygielska ^1^, Jeremy Pike ^1,3^, Samuel Kemble ^4^, Antonio Rodriguez-Romero ^5^, Christina Simoglou Karali ^5^, Adam P. Croft ^4^, Bethan Psaila ^5,6^, Filipa Simões ^2^, Julie Rayes ^1,3^, Abdullah O. Khan *^1,5^

**Supplemental Material**

**
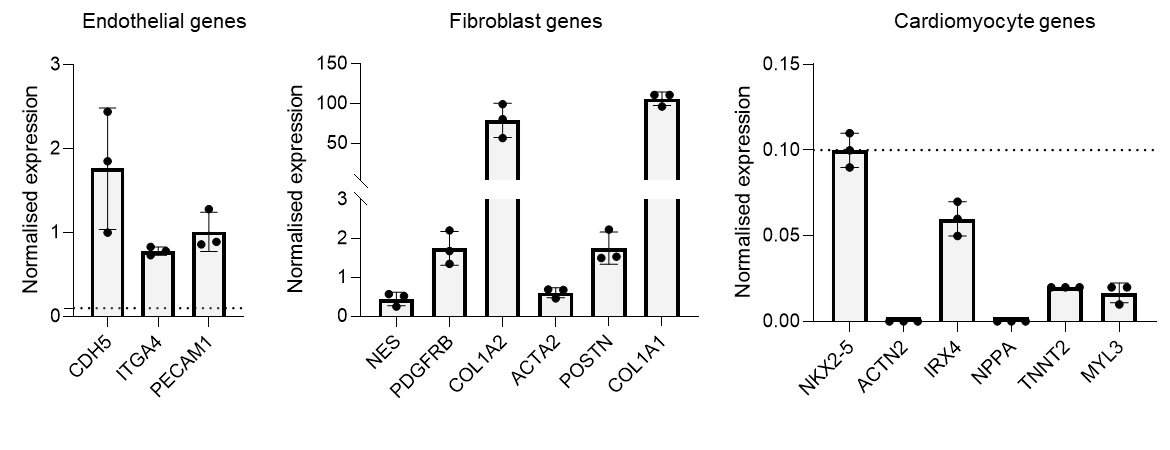
**

**SUPPLEMENTARY FIGURE 1. Gene expression profiling of vascular sprouts.** Expression of endothelial (*CDH5*, *ITGA4* and *PECAM1*), fibroblast *(NES*, *PDGFRβ*, *COL1A2*, *ACTA2*, *POSTN* and *COL1A1*) and cardiomyocyte (*NKX2-5*, *ACTN2*, *IRX4*, *NPPA*, *TNNT2* and *MYL3*) in vascular sprouts. Data are presented as mean ± SD (n = 3 independent experiments) normalised to *GAPDH* expression.


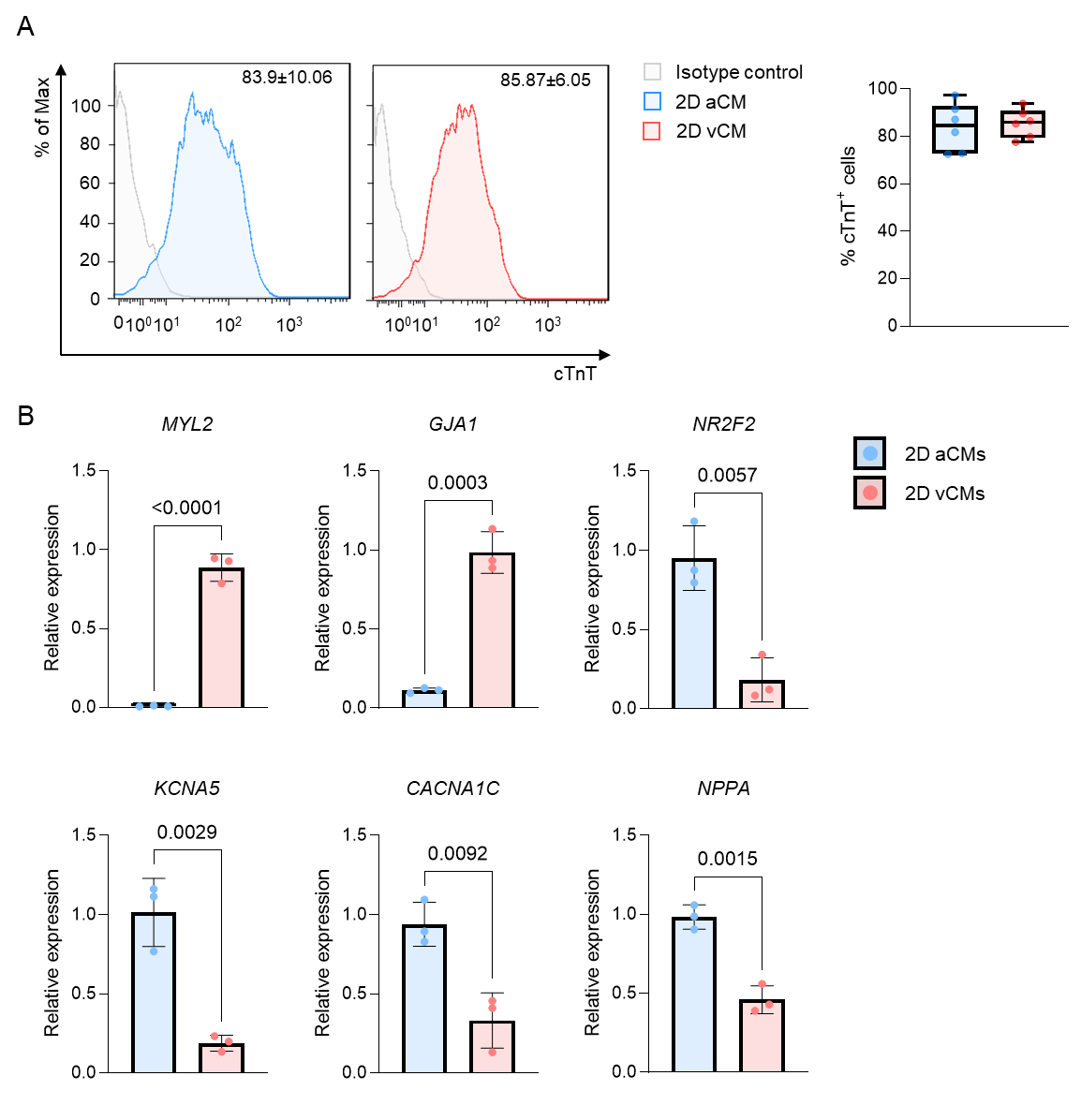


**SUPPLEMENTARY FIGURE 2. Validation of atrial and ventricular cardiomyocyte specificity.** (A) Expression of cardiac troponin-t (cTnT) in 2D derived atrial and ventricular cardiomyocytes as assessed by flow cytometry. Quantification is shown right. (B) Expression of ventricular (*MYL2* and *GJA1*) and atrial (*NR2F2, NPPA, KCNA5* and *CACNA1C*) in 2D hiPSC derived atrial and ventricular cardiomyocytes. Data are presented as mean ± SD (n = 3 independent experiments from 3 separate differentiations) relative to 2D hiPSC derived ventricular cardiomyocytes for the ventricular cardiomyocyte genes *MYL2* and *GJA1* or relative to 2D hiPSC atrial cardiomyocytes for the atrial cardiomyocyte genes for *NR2F2*, *KCNA5*, *CACNA1C* and *NPPA*. Statistical analysis was performed using a Mann-Whitney U-Test.

**
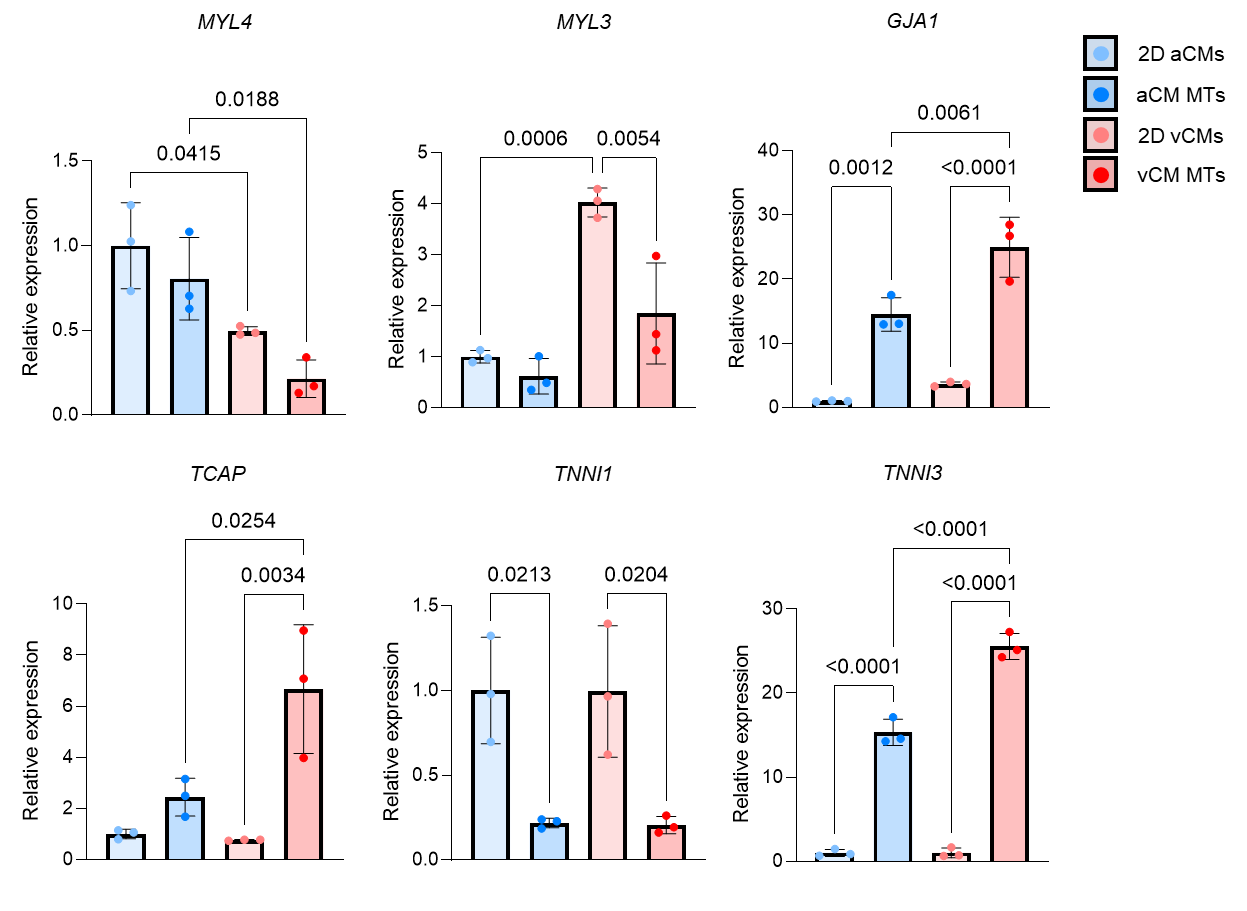
**

**SUPPLEMENTARY FIGURE 3. Gene expression profiling of cardiac structural genes in aCM-MTs and vCM-MTs.** Expression of cardiomyocyte structural genes (*MYL4*, *MYL3*, *GJA1*, *TCAP*, *TNNI1* and *TNNI3*) in 2D hiPSC derived atrial and ventricular cardiomyocytes and 3D aCM MTs and vCM MTs. Data are presented as mean ± SD (n = 3 independent experiments consisting of 3 separate differentiations of 12 pooled cardiac microtissues) relative to 2D hiPSC derived atrial cardiomyocytes. Statistical analysis was performed using a Kruskal-Wallis test.


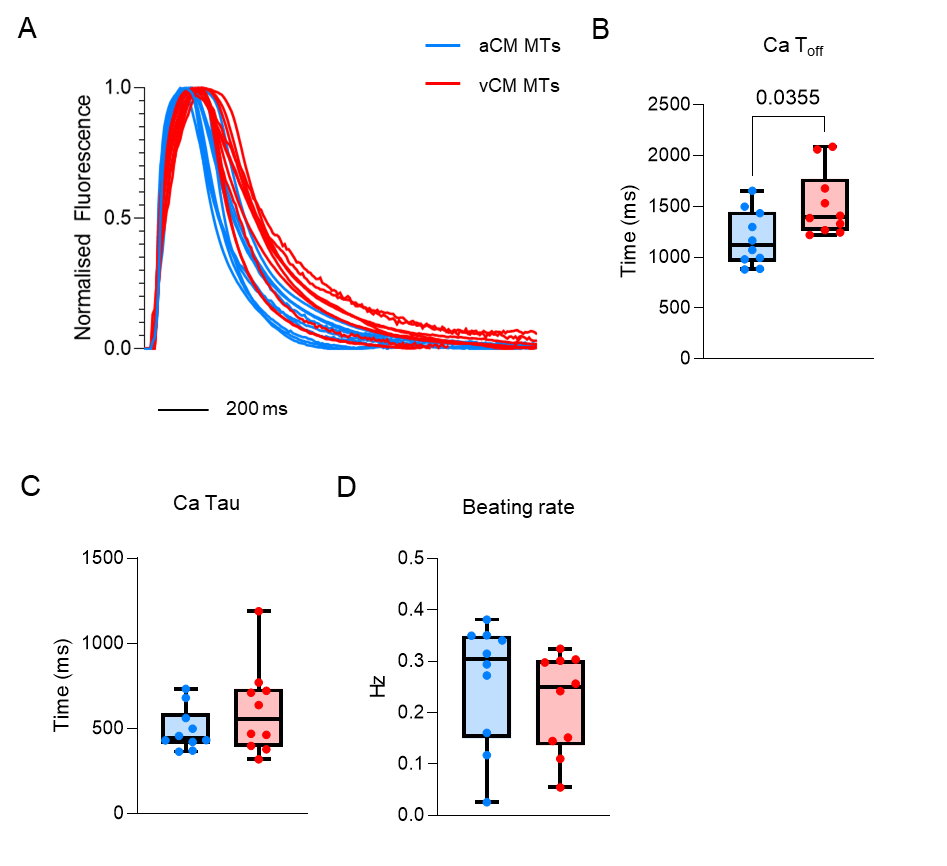


**SUPPLEMENTARY FIGURE 4. Ca^2+^ transient analysis in atrial and ventricular microtissues.** (A) Overlayed normalised Ca^2+^ transients from aCM MTs and vCM MTs. (B) Quantification of time for calcium transient decay (Ca T_off_), (C) calcium decay constant (Ca Tau) and (D) beating rate in aCM MTs and vCM MTs. Data are presented as mean ± SD (n = 10 aCM MTs and n = 10 vCM MTs from 3 independent experiments). Statistical analysis was performed using a Mann-Whitney U-test.

**
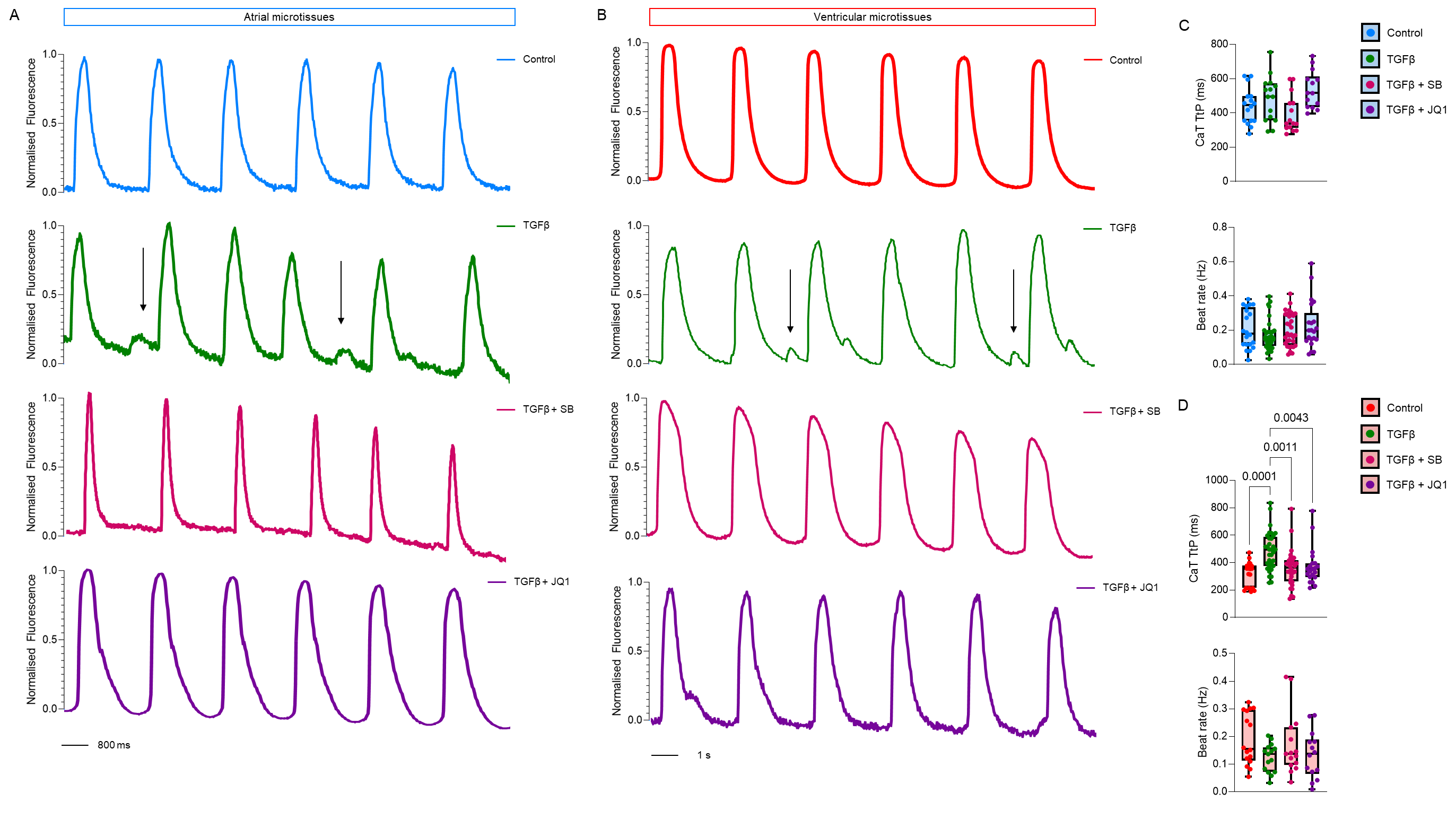
**

**SUPPLEMENTARY FIGURE 5. Additional Ca^2+^ analysis in atrial and ventricular microtissues treated with TGFβ in the presence or absence of the TGFβ inhibitors SB431542 or JQ1.** Representative Ca^2+^ traces of aCM MTs (A) or vCM MTs (B) under control conditions or following treatment with TGFβ in the presence or absence of the TGFβ inhibitor SB431542 (SB) or the BET bromodomain inhibitor JQ1. Arrows represent the presence of ectopic firing events. Quantification of calcium time-to-peak (Ca TtP) and beating rate in (C) aCM MTs and (D) vCM MTs. Data are presented as mean ± SD (n = 20, control, n = 33 TGFβ, n = 32 TGFβ + SB, n = 20 TGFβ + JQ1 treated aCMs or n = 16, control, n = 15 TGFβ, n = 15 TGFβ + SB, n = 14 TGFβ + JQ1) from 4 independent experiments. Statistical analysis was performed using a Kruskal-Wallis test.

**
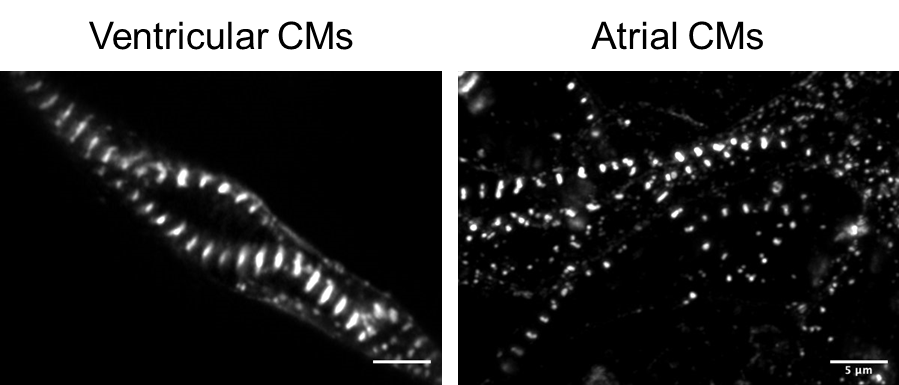
**

**SUPPLEMENTARY FIGURE 6. Assessment of α-actinin imaging in disaggregated atrial and ventricular cardiomyocytes from aCM and vCM MTs.** High resolution representative images of α-actinin staining from aCM’s and vCM’s isolated from disaggregated aCM and vCM MTs. Scale bar = 5µm.

**Supplementary Table 1.** Primer sequences used for qRT-PCR studies

| **Gene** | **Primer Sequence (5’ – 3’)** | | **Annealing Temperature (°C)** |
| --- | --- | --- | --- |
| ACTN2 | FWD | TTC CTA TGG GGT CAT CCT TG | 60 |
|  | REV | CTG CTG CTT TGG TGT CAG AG |  |
| ATP2A2 | FWD | CTC CTC AGG TCA ACC CAT AAA G | 60 |
|  | REV | GTG TCG TCT GGC TAC AGA ATA A |  |
| CACNA1C | FWD | GAG GAA GAG GAG AAG GAG AGA A | 61 |
|  | REV | CAG GTC CAG GAT GTT GAA GTA G |  |
| CDH5 | FWD | GAA ACA GAG CCC AGG TCA TTA T | 60 |
|  | REV | GAT GGT GAG GAT GCA GAG TAA G |  |
| GAPDH | FWD | GAT TCC ACC CAT GGC AAA TTC | 60 |
|  | REV | GTC ATG AGT CCT TCC AGC ATA C |  |
| HEY2 | FWD | GAT TCA GCC CTC CGA ATG | 59 |
|  | REV | TGG CAG AGA GGG ACA AGA G |  |
| IRX4 | FWD | TTC CGT TCT GAA GCG TGG TC | 61 |
|  | REV | TGA AGC AGG CAA TTA TTG GTG T |  |
| KCNA5 | FWD | CGA GGA TGA GGG CTT CAT TA | 60 |
|  | REV | CTG AAC TCA GGC AGG GTC TC |  |
| MYL2 | FWD | GAT GTT CGC CTT CCC CGC | 66 |
|  | REV | GCA GCG AGC CCC CTC CTA GT |  |
| NES | FWD | CAC TCA GAG GGA AGG AGA TAG A | 61 |
|  | REV | GAT GGA GCA GGC AAG AGA TT |  |
| NPPA | FWD | ACA GGA TTG GAG CCC AGA G | 63 |
|  | REV | GGA GCC TCT TGC AGT CTG TC |  |
| NR2F2 | FWD | CCG AGT ACA GCT GCC TCA A | 60 |
|  | REV | TTT TCC TGC AAG CTT TCC AC |  |
| NXK2-5 | FWD | TTC CCG CCG CCC CCG CCT TCT AT | 67 |
|  | REV | CGC TCC GCG TTG TCC GCC TCT GT |  |
| PECAM1 | FWD | CTC TAC ACC CAA GTT CCA CAT C | 61 |
|  | REV | CAC CCT CAG AAC CTC ACT TAA C |  |
| PDGFRB | FWD | GCT CAC CAT CAT CTC CCT TAT C | 60 |
|  | REV | GAG GAC TCG ATG TCT GCA TAT T |  |
| RYR2 | FWD | CCC TAT GGA CCT GAG CTT TAT C | 60 |
|  | REV | CTG TCT ATG GTG CCG TCT ATT C |  |
| TNNI1 | FWD | CTT TAG GGC GTG GGT CTT ATC | 60 |
|  | REV | GTA TGC GTG TCC TGG TTA CTT |  |
| TNNI3 | FWD | TCC AAC TAC CGC GCT TAT G | 60 |
|  | REV | TGC CTC TAT GTC GTA TCT CTC T |  |
| TNNT2 | FWD | TTC GAC CTG CAG GAG AAG TT | 62 |
|  | REV | GCG GGT CTT GGA GAC TTT CT |  |
| **Gene** | **PrimeTime Primer Code** | | |
| ACTA2 | Hs.PT.56a.2542642 | | |
| COL1A1 | Hs.PT.58.15517795 | | |
| COL1A2 | Hs.PT.58.2783065 | | |
| DES | Hs.PT.58.27885793 | | |
| GJA1 | Hs.PT.58.38338544 | | |
| ITAG4 | Hs.PT.58.40415661 | | |
| MYL3 | Hs.PT.58.19762325 | | |
| MYL4 | Hs.PT.58.27527748.g | | |
| POSTN | Hs.PT.58.4452022 | | |
| TCAP | Hs.PT.58.25615104.g | | |

**Supplementary Table 2.** Antibodies and dilutions used for immunofluorescence studies

| **Primary Antibody** | **Dilution** | **Supplier** |
| --- | --- | --- |
| α-actinin, monoclonal mouse IgG1 | 1:1000 | Sigma-Aldrich, clone EA-53 |
| MLC2A, monoclonal mouse IgG2a | 1:200 | Synaptic Systems, clone 56F5 |
| MLC2v, polyclonal rabbit IgG | 1:200 | Proteintech, catalogue 10906-1-AP |
| Ulex Europaeus Agglutinin-1 (UEA1), biotinylated | 1:200 | Vector laboratories, catalogue B-1065-2 |
| PDGFRβ | 1:500 | Abcam, Clone Y92 |
| α-Smooth muscle actin | 1:300 | Sigma Aldrich, Clone 1A4 |
| Collagen 1 | 1:300 | Sigma Aldrich, Clone Col-1 |
| **Secondary Antibody** | **Dilution** | **Supplier** |
| Alexa Fluor 488, polyclonal donkey α-mouse IgG | 1:1000 | Thermo Fisher, catalogue A21202 |
| Alexa Fluor 564, polyclonal donkey α-rabbit IgG | 1:1000 | Thermo Fisher, catalogue A11011 |
| Alexa Fluor 488 Goat Anti-Mouse IgG2a | 1:1000 | Thermo Fisher, catalogue A21131 |
| Alexa Fluor 568 Goat Anti-Mouse IgG1 (y1)a | 1:1000 | Thermo Fisher, catalogue A21124 |
